# Enzyme‐Activatable Chemokine Conjugates for In Vivo Targeting of Tumor‐Associated Macrophages

**DOI:** 10.1002/ange.202207508

**Published:** 2022-09-05

**Authors:** Nicole D. Barth, Floris J. Van Dalen, Utsa Karmakar, Marco Bertolini, Lorena Mendive‐Tapia, Takanori Kitamura, Martijn Verdoes, Marc Vendrell

**Affiliations:** ^1^ Centre for Inflammation Research University of Edinburgh UK; ^2^ Cancer Research UK Edinburgh Centre University of Edinburgh UK; ^3^ Dept. Tumor Immunology and Institute for Chemical Immunology Radboud Institute for Molecular Life Sciences Radboud University Medical Center The Netherlands; ^4^ MRC Centre for Reproductive Health University of Edinburgh UK

**Keywords:** CCL2, Cancer, Cathepsins, Probes, Prodrugs

## Abstract

Increased levels of tumor‐associated macrophages (TAMs) are indicators of poor prognosis in most cancers. Although antibodies and small molecules blocking the recruitment of macrophages to tumors are under evaluation as anticancer therapies, these strategies are not specific for macrophage subpopulations. Herein we report the first enzyme‐activatable chemokine conjugates for effective targeting of defined macrophage subsets in live tumors. Our constructs exploit the high expression of chemokine receptors (e.g., CCR2) and the activity of cysteine cathepsins in TAMs to target these cells selectively over other macrophages and immune cells (e.g., neutrophils, T cells, B cells). Furthermore, we demonstrate that cathepsin‐activatable chemokines are compatible with both fluorescent and therapeutic cargos, opening new avenues in the design of targeted theranostic probes for immune cells in the tumor microenvironment.

## Graphical abstract

We describe the first enzyme‐activatable chemokine conjugates for selective targeting of tumor‐associated macrophages in preclinical models of cancer. We demonstrate that this new chemical design is compatible with both fluorescent and therapeutic payloads, opening avenues in the preparation of future theranostic probes for immune cells in the tumor microenvironment.

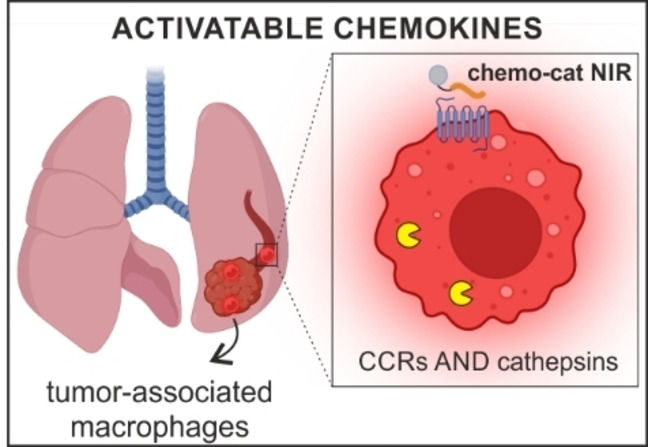

## Introduction

Macrophages are critical regulators of tissue homeostasis,^[1]^ but some subpopulations (e.g., tumor‐associated macrophages, TAMs) support the growth, angiogenesis and metastasis of tumors.^[2]^ TAMs suppress anticancer immune responses to accelerate the intravasation and spreading of tumor cells; therefore, the infiltration of TAMs in tumors correlates with poor prognosis in most cancers.^[3]^ Because complete macrophage depletion is not therapeutically sustainable for prolonged periods of time,^[4]^ strategies to inhibit macrophages with antibodies (e.g., anti‐CSF1R)^[5]^ or small molecules (e.g., bisphosphonates)^[6]^ have been designed. However, there are very few chemical structures to target TAMs in live tumors with good selectivity over other macrophages (e.g., tissue‐resident macrophages) and immune cells (e.g., neutrophils, T cells and B cells).

One of the main mechanisms that cancer cells employ to increase the recruitment of TAMs to primary tumors and metastatic sites is the release of monocyte chemoattractant protein 1 (MCP‐1 or CCL2),^[7]^ which attracts monocytes to the tumor microenvironment (TME) through the CCL2‐CCR2 cascade.^[8]^ Once monocytes reach tumor sites, they differentiate into TAMs to support the expansion and intravasation of cancer cells. Furthermore, TAMs are known to upregulate the expression of cysteine cathepsins,^[9]^ which 1) facilitates further recruitment of monocytes from circulation to the TME,^[10]^ and 2) protects tumors from the effect of some chemotherapeutic drugs.^[9]^ Fluorescent probes including cell‐targeting peptide structures^[11]^ or chemokine proteins^[12]^ as well as cathepsin‐reactive activity‐based probes^[13]^ have been used to image cancer models, yet there are no examples of cathepsin‐activatable chemokines as dual‐selective AND‐gate probes to target subsets of macrophages in tumors.

**Scheme 1:**
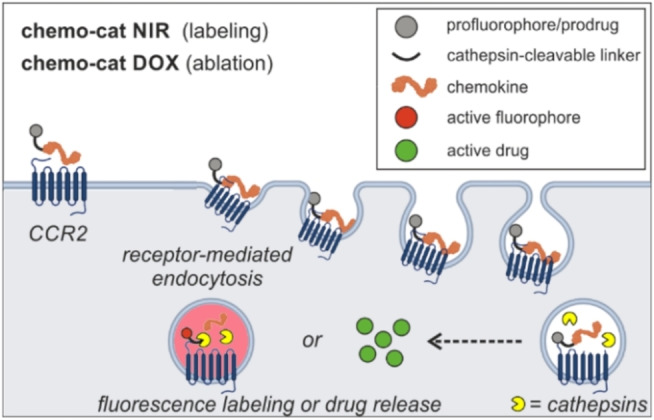
Enzyme‐activatable chemokine conjugates targeting TAMs. Chemokine constructs enter TAMs through CCR2‐mediated endocytosis where they undergo cathepsin‐dependent activation for near‐infrared fluorescence labeling (fluorescent probe **chemo‐cat NIR**) or doxorubicin‐mediated cell ablation (prodrug **chemo‐cat DOX**), respectively.

Antibody‐drug conjugates, which use monoclonal antibodies for cell targeting, have been successfully translated to the clinic;^[14]^ however, antibodies for some proteins (e.g., chemokine receptors) might be not readily available or difficult to generate. Chemical constructs that rely on more than one biomarker (e.g., AND‐gates,[^12a^, ^15^, ^16^] dual‐locked probes^[17]^) can maximize cell specificity as well as multiplexed detection. Their application is particularly favorable when the recognition of the molecular targets is sequential (i.e., receptor‐mediated internalization followed by intracellular enzymatic activation, Scheme 1) because it leads to very low background signals and enables the distinction of closely related populations of cells. In this work, we targeted two biomarkers of TAMs (i.e., CCR2 expression AND cysteine cathepsin activity) to construct some of the first activatable chemokines for TAMs (Scheme 1). We demonstrate that this chemical strategy is versatile and compatible with near‐infrared (NIR) fluorophores^[18]^ and therapeutic payloads for the optical detection as well as the ablation of TAMs in preclinical models of metastatic breast cancer.

## Results and Discussion

We first designed **chemo‐cat NIR** as a NIR fluorescent probe to image TAMs by exploiting a sequential activation mechanism, whereby the probe would 1) enter cells via CCR2‐mediated endocytosis and 2) undergo fluorophore activation by reaction with intracellular cysteine cathepsins (Scheme 1). We envisioned that a molecular AND‐gate design could lead to selective labeling of CCR2+ TAMs containing active cysteine cathepsins, but not any other cells found in the TME.

**Figure 1:**
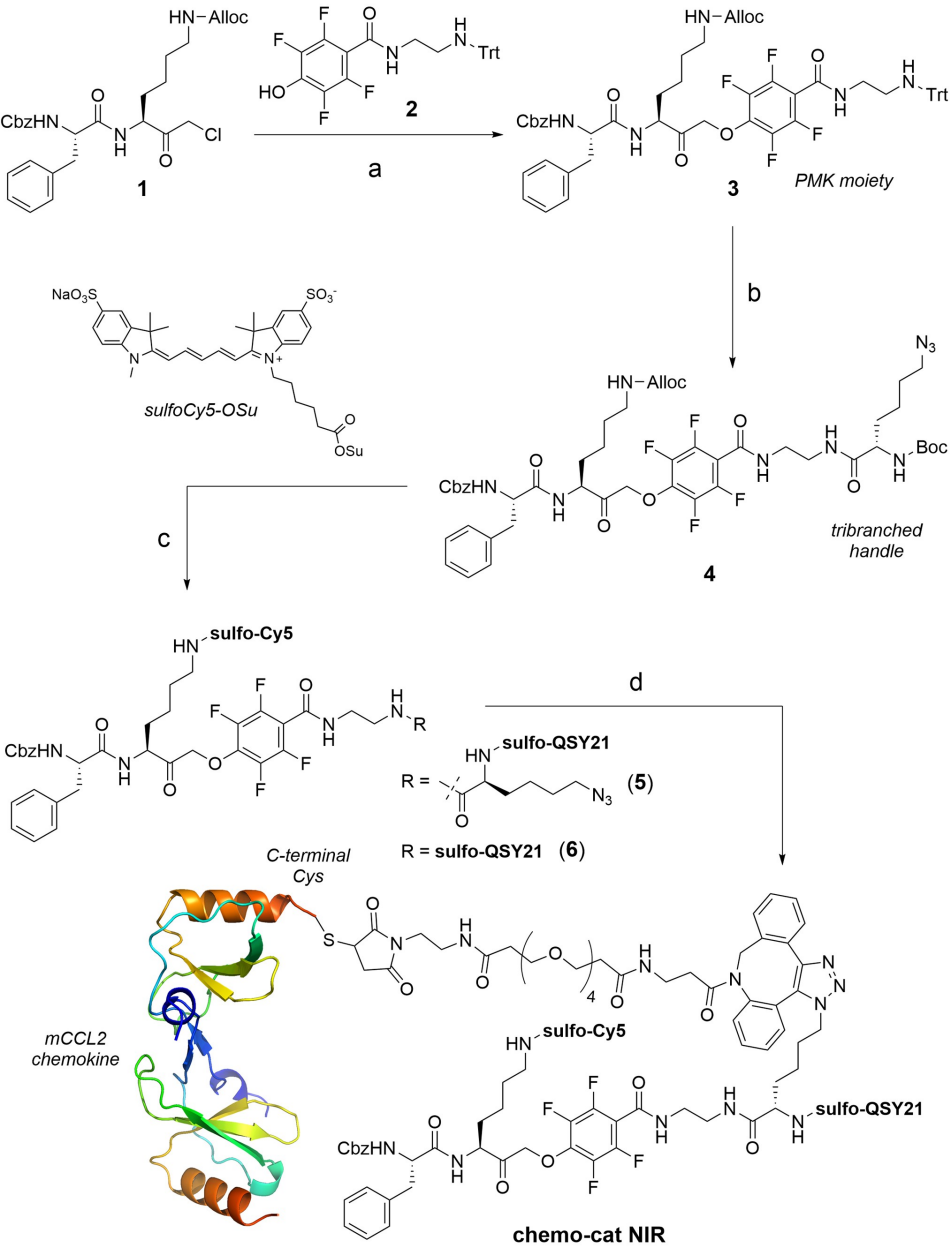
Synthesis of the fluorescent activatable probe **chemo‐cat NIR**. Reagents and conditions: a) compound **2**, KF, DMF, 60 °C, 16 h, b) i) TFA/TIS/DCM, r.t., 30 min, ii) Boc‐L‐Lys(azido)‐OSu, DIPEA, DMF, r.t., 16 h, 94 % for 3 steps; c) i) Pd(PPh_3_)_4_, DMBA, THF, r.t., 10 min, ii) Sulfo‐Cy5‐OSu, DIPEA, DMSO, r.t., 16 h, iii) TFA/DCM, r.t., 30 min, iv) Sulfo‐QSY21‐OSu, DIPEA, DMSO, r.t., 16 h, 21 % for 4 steps; d) i) DBCO‐PEG4 maleimide, DMSO, r.t., 16 h, 54 %, ii) mCCL2‐SH, pH 6.5, r.t., 2 h, 30 %. The representation of mCCL2 dimer was prepared with PyMOL Molecular Graphics System V 1.8.2.0 from the PDB file 1DOK (2.4 Å).

CCL2 is a 9 kDa protein that acts as the native ligand of CCR2^[19]^ with high selectivity over other G‐protein coupled receptors that are expressed on immune cells. Recent studies have identified high levels of CCR2 at the surface of TAMs.^[20]^ Because CCR2 binding is primarily mediated by the N‐terminal domain of CCL2,^[21]^ we prepared **chemo‐cat NIR** by conjugating a cathepsin‐cleavable linker and a fluorophore:quencher pair (i.e., sulfo‐Cy5 as the fluorophore and sulfo‐QSY21 as the quencher) to the C‐terminal end of mouse CCL2 (mCCL2) so that binding of the chemokine to mouse CCR2 (mCCR2) was retained. As the linker we employed a cathepsin‐cleavable phenoxymethyl ketone (PMK, Figure 1), which has been previously described as a warhead in activity‐based probes for cysteine cathepsins.^[22]^ We positioned a conjugation handle on the leaving group of the PMK moiety such that the fragment containing mCCL2 and the sulfo‐QSY21 quencher would be cleaved off after nucleophilic attack on the PMK by cysteine cathepsins to covalently attach and activate the sulfo‐Cy5 fluorophore. This chemical design would allow us to directly compare the protein labeling profiles of the probe **chemo‐cat NIR** and its chemokine‐free analogue (**6**, Figure 1) by SDS‐PAGE analysis as the fluorescent conjugates resulting from the covalent coupling to cysteine cathepsins would be identical for these two compounds.

We started the synthesis of **chemo‐cat NIR** with the preparation of the chloromethyl ketone **1**, which was reacted with the trityl‐protected tetrafluorophenol derivative **2** to obtain the PMK compound **3** (Figure 1). After removal of the trityl group under mild acidic conditions, we introduced Boc‐l‐Lys(azido)‐OSu as a tribranched handle to enable coupling to mCCL2 as well as introduction of the quencher (**4**, Figure 1). Next, we used orthogonal deprotection steps to derivatize the two Lys residues with a sulfo‐Cy5 fluorophore or a sulfo‐QSY21 quencher to render compound **5** (Figure 1). The azide was reacted with a dibenzocyclooctyne (DBCO)‐functionalized PEG maleimide spacer for site‐specific coupling to Cys‐derivatized mCCL2. With this approach, we aimed to minimize any potential steric hindrance between the cathepsin‐reactive moiety and the chemokine. Lastly, **chemo‐cat NIR** was isolated in high purity (>90 %) by conjugation to mCCL2‐thiol in aqueous buffer at pH 6.5 and purification by HPLC (see Electronic Supporting Information for synthetic and characterization details).

**Figure 2:**
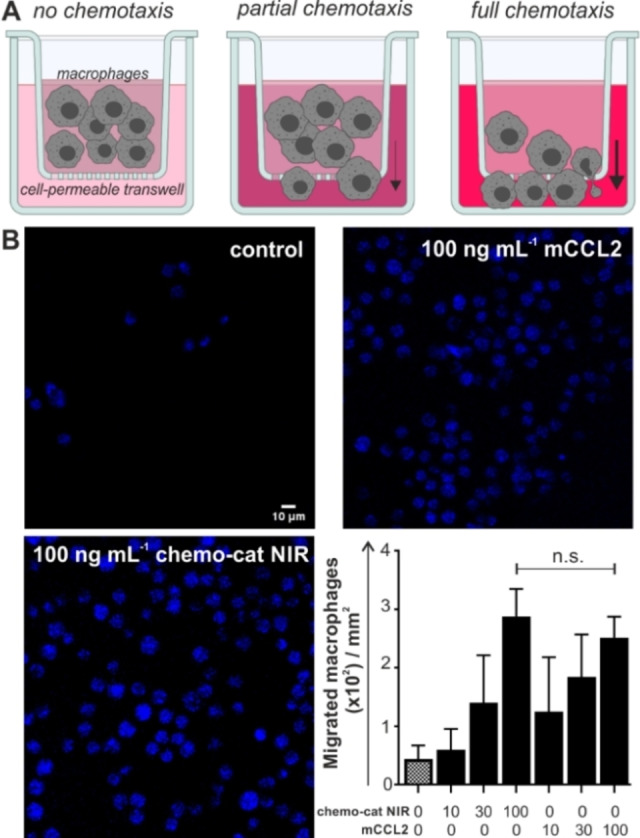
**Chemo‐cat NIR** retains chemotactic activity in mouse macrophages. A) Schematic illustration of transwell assays in RAW264.7 macrophages where migrated cells were stained with Hoechst 33342 after 2 h incubation with media containing increasing concentrations of unlabeled mCCL2 or **chemo‐cat NIR** (0, 10, 30 and 100 ng mL^−1^). B) Representative fluorescence microscopy images (from *n*=3) of Hoechst‐stained migrated macrophages after incubation with mCCL2 or **chemo‐cat NIR**. Scale bar: 10 μm. Cell counting was performed using ImageJ and values are presented as means±SD (*n*=3). P values were determined using one‐way ANOVA (n.s. for *p*>0.05).

After the synthesis of **chemo‐cat NIR**, we examined its recognition properties for CCR2 and cysteine cathepsins. CCL2 is a powerful chemoattractant, therefore we performed transwell assays to determine whether **chemo‐cat NIR** retained the chemotactic ability of mCCL2. For these experiments, we cultured murine RAW264.7 macrophages on cell‐permeable membranes to count the cells responding to the chemokine gradient and migrating to the bottom of the well (Figure 2A). The results indicated that **chemo‐cat NIR** retained the chemotactic activity of unlabeled mCCL2 and we did not observe significant differences between them (Figure 2B). In both cases, we detected elevated levels of migration as the chemokine gradients were increased up to 100 ng mL^−1^. These results confirmed that the C‐terminal derivatization of mCCL2 with the cathepsin‐targeting group did not impair the biological function of the chemokine. The spectral characterization of **chemo‐cat NIR** also corroborated that its photophysical profile resembled that of sulfo‐Cy5 (λ_exc_: 650 nm, λ_em_: 670 nm, Figure S1), making it compatible with most flow cytometers and confocal microscopes.

**Figure 3:**
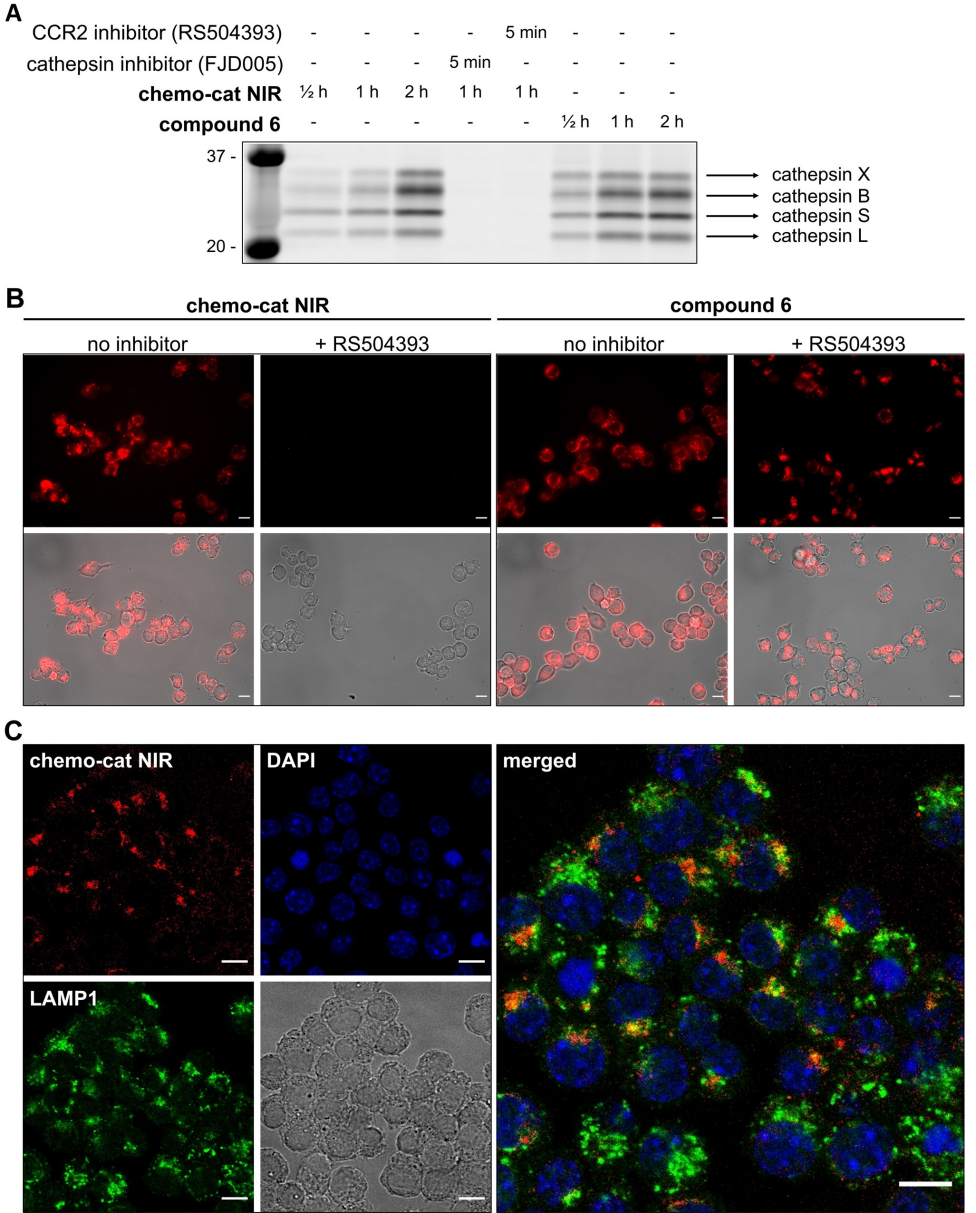
**Chemo‐cat NIR** enters macrophages in a CCR2‐dependent manner and is cleaved by intracellular cathepsins. A) RAW264.7 macrophages were pre‐incubated or not with the inhibitors (RS504393 or FJD005, both 2.5 μM for 5 min) followed by labeling with **chemo‐cat NIR** or compound **6** (250 nM) at 37 °C. Cells were lysed and proteins were separated by SDS‐PAGE and visualized by in‐gel fluorescence scanning. B,C) Representative fluorescence and brightfield microscopy images (from *n*=3) of RAW264.7 macrophages after incubation with **chemo‐cat NIR** or compound **6** (red, 250 nM) with or without treatment of RS504393 (B) and co‐staining with anti‐LAMP1 (green) and DAPI (blue) (C). Scale bars: 10 μm.

Next, we evaluated the capacity of **chemo‐cat NIR** to label live macrophages in a CCR2 and cathepsin‐dependent manner (Figure S2). First, we confirmed that CCR2 receptors are expressed on the macrophage cell line RAW264.7 using commercially available anti‐CCR2 antibodies (Figure S3). Then, we analyzed the labeling of live RAW264.7 macrophages upon incubation with **chemo‐cat NIR** or its analogue **6**. We compared the labeling profiles by SDS‐PAGE analysis of the lysates and observed that both compounds reacted to a similar extent with intracellular cathepsins (Figure 3A). These results confirmed that the chemokine mCCL2 did not impair the reactivity of the cathepsin‐cleavable linker. Importantly, we also performed experiments where we pre‐treated macrophages with the CCR2 antagonist RS504393 or the cathepsin inhibitor FJD005^[23]^ before incubation with **chemo‐cat NIR** (Figure 3A). In both cases, we observed a dramatic reduction in fluorescence labeling, indicating that the staining by **chemo‐cat NIR** was dependent on CCR2‐mediated endocytosis and cathepsin activity. The protein gels highlighted that, like its parent analogue **6**, **chemo‐cat NIR** is a pan‐reactive cysteine cathepsin reporter as shown by the formation of fluorescent conjugates with cathepsin X, B, S and L (Figure 3A).

We also examined the application of **chemo‐cat NIR** in live‐cell fluorescence microscopy. We observed activation of **chemo‐cat NIR** fluorescence in intracellular compartments in RAW264.7 macrophages. Notably, the emission was completely abolished by pretreatment with the CCR2 antagonist RS504393, unlike for the chemokine‐free analogue **6**, corroborating that **chemo‐cat NIR** enters macrophages via CCR2‐mediated transport (Figure 3B). Confocal microscopy experiments showed that **chemo‐cat NIR** is predominantly localized in intracellular vesicles and activated in both early and late endosomal compartments as demonstrated by the partial co‐staining with Lysosomal Associated Membrane Protein 1 (LAMP1, Figure 3C).

Finally, we performed flow cytometry experiments to confirm the cathepsin‐dependent activation of **chemo‐cat NIR** in live macrophages. The fluorescence labeling of macrophages was significantly reduced by pre‐treatment with the cathepsin inhibitor FJD005 (Figure S4). We compared **chemo‐cat NIR** to the commercial chemokine AF647‐mCCL2, where the fluorophore AlexaFluor647 (AF647) does not require cathepsin activation. In contrast to **chemo‐cat NIR**, AF647‐mCCL2 displayed fluorescent signals even in the presence of the cathepsin inhibitor FJD005 (Figure S5). Altogether, these results show that **chemo‐cat NIR** enters macrophages via CCR2‐mediated endocytosis and emits fluorescence after activation by intracellular cathepsins.

**Figure 4:**
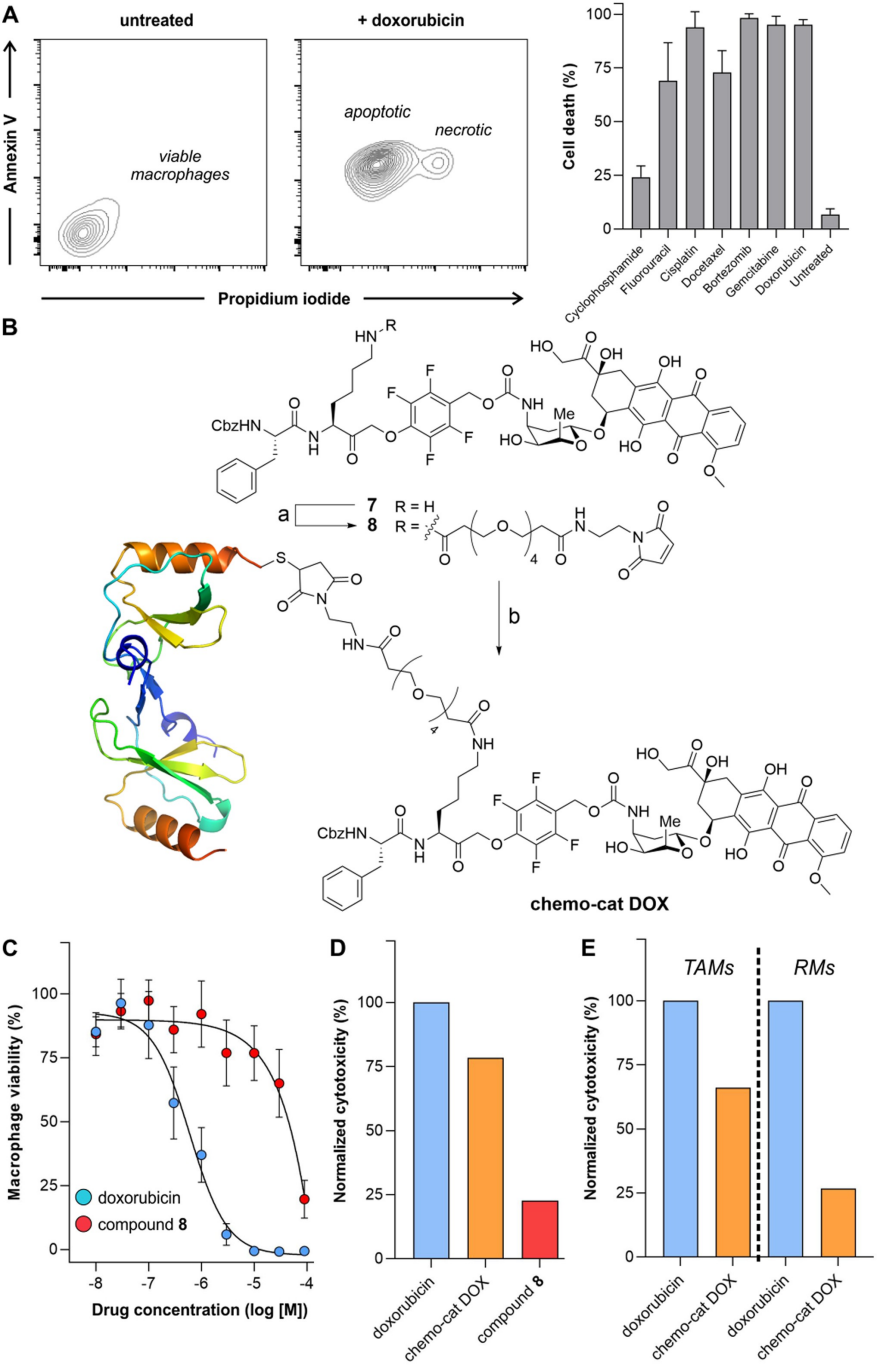
**Chemo‐cat DOX** as a prodrug for ablation of TAMs. A) Representative flow cytometry contour plots of viable macrophages (untreated) as well as doxorubicin‐treated apoptotic (Annexin V+ PI−) and necrotic (Annexin V+ PI+) macrophages. Percentages of cell death in RAW264.7 macrophages after incubation with the indicated drugs (10 μM, 24 h) at 37 °C. Cells were stained with AF647‐Annexin V (25 nM) and PI (3 μM) before analysis by flow cytometry. Data presented as means±SD (*n*=3). B) Reagents and conditions: a) compound **7**, OSu‐PEG4 maleimide, DIPEA, DMSO, r.t., 16 h, 81 %, b) mCCL2‐SH, pH 6.5, r.t., 2 h, 20 %. C) Dose response curves of doxorubicin and compound **8** in RAW264.7 macrophages. Values presented as means±SD (*n*=3). D,E) Normalized toxicity (relative to doxorubicin alone) in RAW264.7 macrophages (D) or in TAMs and RMs isolated from tumor‐bearing mouse lungs (E) after incubation with doxorubicin, compound **8** and **chemo‐cat DOX** (all at 1 μM for 48 h). Values presented as means from 2 independent experiments.

To explore whether activatable chemokines would enable the delivery of therapeutic payloads in macrophages, we constructed a chemokine‐based prodrug to selectively ablate recruited TAMs over tissue‐resident macrophages. First, we screened the cytotoxic capacity of several FDA‐approved drugs in RAW264.7 macrophages, namely DNA replication inhibitors,[^24^, ^25^, ^26^, ^27^] topoisomerase inhibitors,^[28]^ proteasomal inhibitors^[29]^ and mitotic inhibitors^[30]^ (Table S1). We incubated the macrophages with seven different drugs and determined the total of apoptotic and necrotic cells after 24 h using an Annexin V/propidium iodide (PI) assay by flow cytometry (Figure 4A). The four most potent molecules (i.e., doxorubicin, bortezomib, cisplatin, gemcitabine) were selected for a dose‐response study, from which doxorubicin was shown to rapidly induce apoptosis in a dose‐dependent manner (Figure S6). Therefore, we decided to synthesize **chemo‐cat DOX** (Figure 4B) as a new derivative where we coupled mCCL2‐thiol to a cathepsin‐activatable analogue of doxorubicin for selective ablation of TAMs.

The chemical synthesis of **chemo‐cat DOX** was designed in a similar manner to the fluorescent probe **chemo‐cat NIR**. Briefly, a cathepsin‐reactive PMK warhead was conjugated to doxorubicin via a self‐immolative carbamate bond, and the ϵ‐amino group of the Lys residue was coupled to the PEG maleimide spacer for site‐specific protein conjugation (Figure 4B). The resulting cathepsin‐activatable prodrug **8** was isolated in high purity (>95 %) (synthetic and characterization details in the Electronic Supporting Information). Prior to the conjugation of compound **8** to mCCL2, we compared the cytotoxic potential of doxorubicin and the non‐targeted prodrug **8** in RAW264.7 macrophages. We performed dose‐response curves for both compounds, and we observed stronger cytotoxicity for doxorubicin than for compound **8** (Figure 4C). This is likely due to the poorer cell uptake of compound **8** relative to doxorubicin because of the presence of the hydrophilic PEG spacer. Next, we conjugated compound **8** to mCCL2‐thiol to produce **chemo‐cat DOX** as an activatable prodrug to be selectively taken up via CCR2 endocytosis. After the synthesis, we compared the cytotoxic ability of an equimolar amount of doxorubicin, **chemo‐cat DOX** and non‐targeted compound **8** in RAW264.7 macrophages (Figure 4D). Notably, the prodrug **chemo‐cat DOX** killed more cells than its chemokine‐free precursor, which suggests that CCR2‐mediated internalization facilitates the delivery of the caged doxorubicin to macrophages.

The targeted delivery of drugs into specific subpopulations of macrophages (e.g., TAMs) represents an attractive approach to minimize the potential side effects of cytotoxic molecules.^[31]^ Several approaches including pH and enzyme‐activatable prodrugs have been reported,^[32]^ yet there are no examples of chemokine‐based constructs including therapeutic payloads to target TAMs. Because cancer‐promoting TAMs express higher levels of CCR2 and cysteine cathepsins than other macrophages, we explored whether **chemo‐cat DOX** could preferentially kill TAMs over tissue‐resident macrophages (RMs), and compared their ex vivo cytotoxicity in these two subpopulations.

Briefly, we used a mouse model in which cancer cells were injected into the tail vein of C57BL/6 mice to grow tumors in the lungs. Two weeks after injection, tumors were detected by bioluminescence imaging (Figure S7) and we harvested the lungs to isolate both TAMs and RMs (Figure S8). Cells were plated and cultured in the presence of doxorubicin or **chemo‐cat DOX** (both at 1 μM) followed by viability analysis after 48 h. Doxorubicin exerted the same cytotoxic effect in all cells, whereas **chemo‐cat DOX** induced more cell death in TAMs than in RMs (Figure 4E). These results indicate that the conjugation of cathepsin‐activatable prodrugs to chemokines (e.g., mCCL2) represents an effective strategy to deliver cytotoxic payloads to tumor‐promoting macrophages that express high levels of chemokine receptors (e.g., mCCR2).

**Figure 5:**
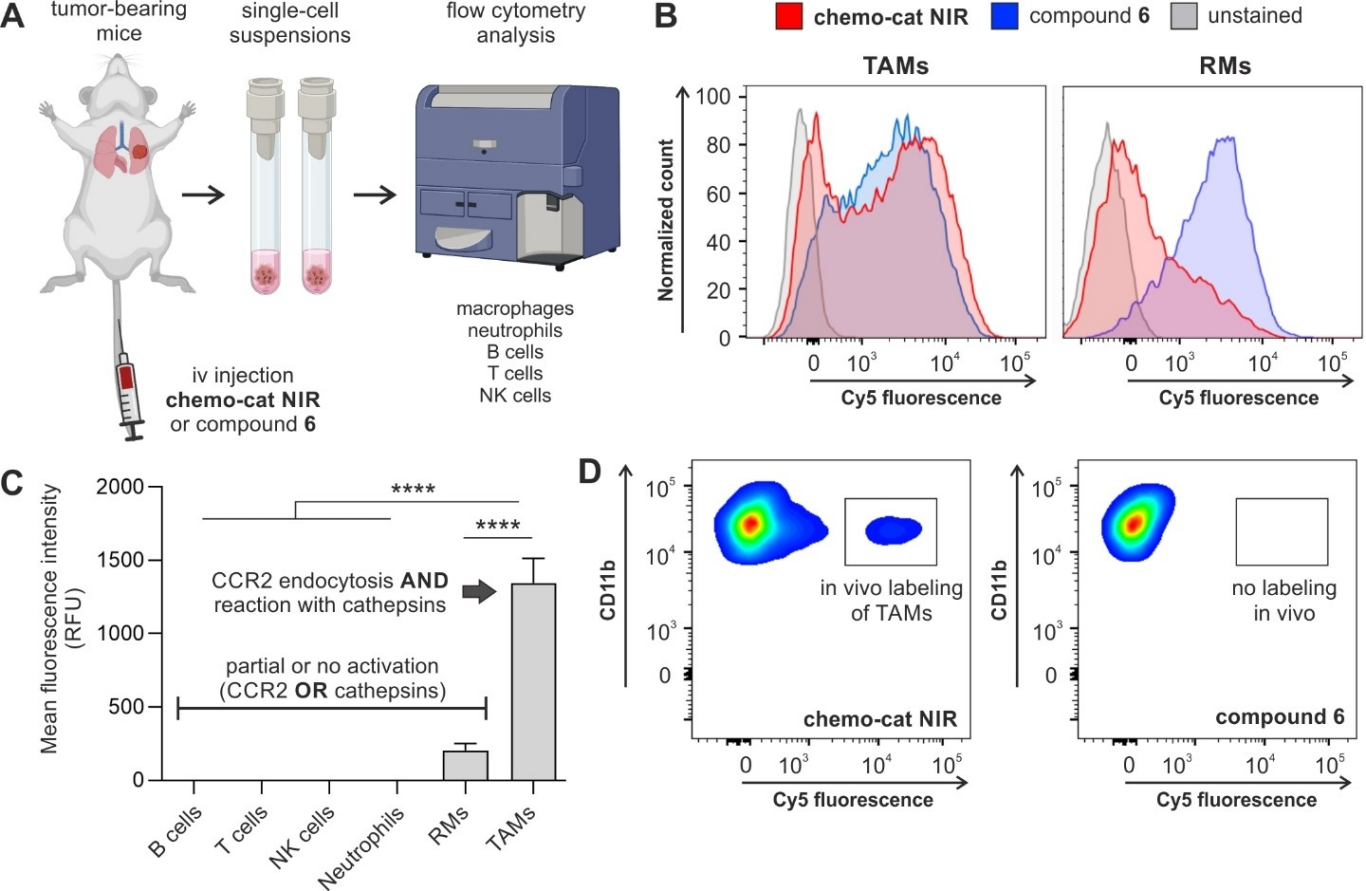
**Chemo‐cat NIR** preferentially labels TAMs in vivo over other macrophages and immune cells. A) Schematic representation of in vivo experiments to evaluate the fluorescence labeling of immune cells by the probe **chemo‐cat NIR** in a mouse model of metastatic breast cancer. B) Single cell suspensions from metastatic lungs (14 days after tail vein injection of E0771‐LG cells) were incubated with **chemo‐cat NIR** or compound **6** (both at 500 nM) for 1 h at 37 °C and analysed by flow cytometry. Representative histograms of TAMs and lung RMs after the incubation of whole‐lung single cell suspensions with **chemo‐cat NIR** (red) or compound **6** (blue) or unstained (grey) (*n*=3). C) Mean fluorescence intensities of different immune cell populations after in vivo tail vein administration of **chemo‐cat NIR** in E0771‐LG tumor‐bearing mice. Neutrophils: F4/80^−^Ly6G^+^, tumor‐associated macrophages and precursor cells (TAMs), F4/80^+^Ly6G^−^CD11c^Low^CD11b^+^, resident macrophages (RMs): F4/80^+^Ly6G^−^CD11c^High^CD11b^Low^Ly6C^Low^, T cells: F4/80^−^Ly6G^−^CD3^+^NK1.1^−^, NK cells; F4/80^−^Ly6G^−^CD3^−^NK1.1^+^, B cells: F4/80^−^Ly6G^−^CD3^−^NK1.1^−^CD19^+^. Data presented as means±SD (*n*=3). P values were determined using one‐way ANOVA (**** for *p*<0.0001). D) Representative flow cytometry plots featuring in vivo fluorescence labeling of TAMs after tail vein administration. Labeling was observed for **chemo‐cat NIR** (red) but not for compound **6** (blue) (both administered at 0.3 nmol per mouse) (*n*=3).

Finally, we investigated whether activatable chemokines could selectively detect TAMs in vivo in a mouse model of metastatic breast cancer.^[33]^ In this model, the breast cancer cells E0771‐LG were injected via tail vein into syngeneic C57BL/6 mice to form metastatic tumors in the lungs within 14 days after injection (Figure 5A). First, we confirmed that the iv administration of **chemo‐cat NIR** did not induce any cytotoxicity or altered the immune cellular composition in vivo (Figures S9 and S10).

Next, we examined the selectivity of **chemo‐cat NIR** for TAMs among other immune cells found in the TME. For this experiment, we harvested tumor‐bearing mouse lungs and obtained single‐cell suspensions that were incubated with **chemo‐cat NIR** or compound **6** (both at 500 nM) for 1 h (Figure 5A). We performed multiparametric flow cytometry to analyse the fluorescent signals from multiple immune cells, namely T cells, NK cells, B cells, neutrophils, lung RMs and TAMs (for detailed antibody panels and gating strategy, see Figure S11). In this analysis, we observed that **chemo‐cat NIR** brightly labeled TAMs but no other immune cells, including lung RMs (Figures 5B,C). On the contrary, the chemokine‐free compound **6** did not distinguish between TAMs, RMs or neutrophils (Figure S12). These results highlight the utility of CCR2 targeting as an effective strategy to selectively deliver activatable fluorophores to TAMs.

Finally, we also assessed whether **chemo‐cat NIR** could label TAMs in vivo using the same preclinical model. Following the formation of metastatic tumors in the lungs, we injected **chemo‐cat NIR** or the non‐targeted analogue **6** (both as intravenous injections, 0.3 nmol per mouse) and harvested lung tissues 1 h after injection. The flow cytometry analysis showed that no labeling of CD11b+ macrophages was detected after injection of compound **6**, whereas the probe **chemo‐cat NIR** brightly labeled TAMs in vivo (Figure 5D). Notably, **chemo‐cat NIR** did not label any other immune cells in lung tumors (Figure S13). The analysis of blood samples from injected mice showed that **chemo‐cat NIR** also labeled circulating monocytes (i.e., the precursor cells of TAMs), in agreement with the high expression of mCCR2 in these cells.^[34]^ Altogether, our results demonstrate that **chemo‐cat NIR** has good biodistribution in vivo and reaches the lungs to selectively label TAMs in mouse models of cancer. The comparison with chemokine‐free fluorophores under the same experimental conditions demonstrates that CCR2‐mediated internalization is a key step towards enhanced selectivity for subpopulations of macrophages that promote cancer progression.

## Conclusion

In this work we prepared the first enzyme‐activatable chemokine conjugates for targeting TAMs in mouse models of cancer. The constructs were synthesized by conjugation of mCCL2‐thiol to cathepsin‐activatable fluorophores (**chemo‐cat NIR**) or caged prodrugs (**chemo‐cat DOX**). Using in vitro assays, we demonstrated that these probes enter macrophages via CCR2‐mediated endocytosis to react with intracellular cysteine cathepsins. **Chemo‐cat NIR** fluorescently labels active cathepsins in macrophages, whereas **chemo‐cat DOX** releases the cytotoxic drug doxorubicin for macrophage ablation. Our results also show that **chemo‐cat NIR** and **chemo‐cat DOX** selectively target TAMs ‐which express high levels of CCR2 and cysteine cathepsins‐ over resident macrophages and other immune cells found in tumors (e.g., neutrophils, T cells, B cells). These dual‐selective probes are complementary to existing antibody‐drug conjugates and will create opportunities for targeting disease‐related subpopulations of immune cells in the tumor microenvironment.

## Conflict of interest

The authors declare no conflict of interest.

1

## Supporting information

As a service to our authors and readers, this journal provides supporting information supplied by the authors. Such materials are peer reviewed and may be re‐organized for online delivery, but are not copy‐edited or typeset. Technical support issues arising from supporting information (other than missing files) should be addressed to the authors.

Supporting Information

## Data Availability

The data that support the findings of this study are available from the corresponding author upon reasonable request.
