# Supplementary material for: Enzyme‐Activatable Chemokine Conjugates for In Vivo Targeting of Tumor‐Associated Macrophages
Source: Angew Chem Weinheim Bergstr Ger. 2022 Sep 5;134(41):e202207508. doi: 10.1002/ange.202207508 (PMC10946784; doi:10.1002/ange.202207508)
Supplement: Supplementary file 1 — Supporting Information [file ANGE-134-0-s001.pdf]

Angewandte Chemie
Eine Zeitschrift der Gesellschaft Deutscher Chemiker

Supporting Information

Enzyme-Activatable Chemokine Conjugates for In Vivo Targeting of Tumor-Associated Macrophages

N. D. Barth, F. J. Van Dalen, U. Karmakar, M. Bertolini, L. Mendive-Tapia, T. Kitamura, M. Verdoes, M. Vendrell**

Electronic Supporting Information

Table of Contents

1. Supplementary Figures
2. Experimental Methods
3. NMR Spectra
4. Supplementary References

Supplementary Figures

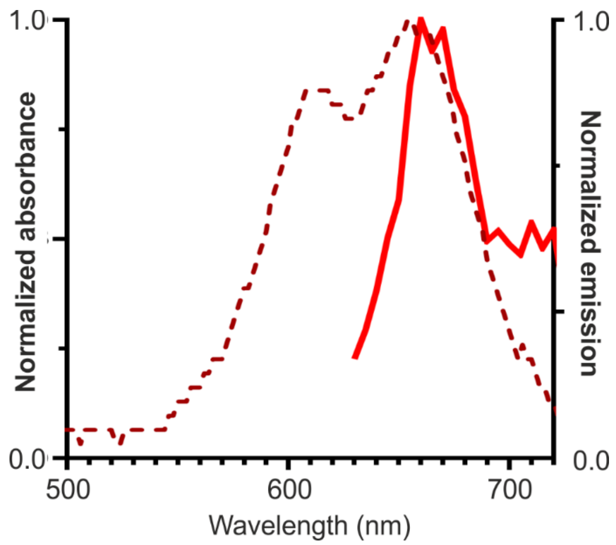

Figure S1. Absorption (dashed line) and emission (solid line) spectra of **chemo-cat NIR** (25 μM) in deionized H_2O, in agreement with the absorbance profiles of sulfo-Cy5 and sulfo-QSY21.

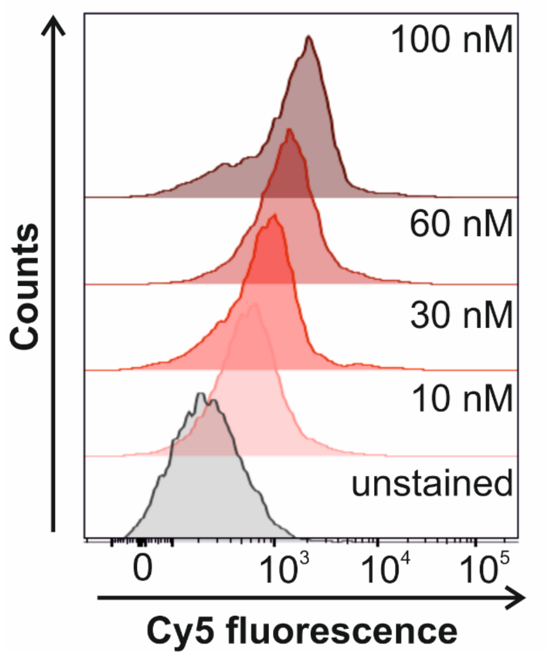

Figure S2. Concentration-dependent fluorescence labeling of RAW264.7 macrophages with **chemo-cat NIR**. Representative histograms after incubation with the indicated concentrations of **chemo-cat NIR** for 1 h at 37°C (n=3).

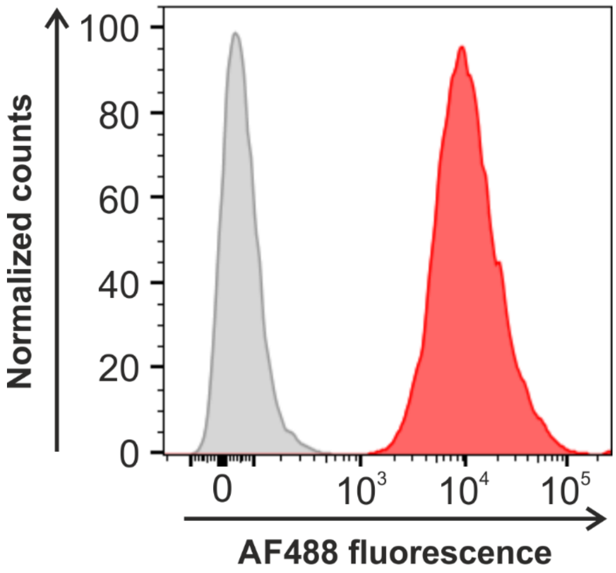

Figure S3. Expression of CCR2 in RAW264.7 macrophages. Representative histograms after incubation of cells with AF488-anti-CCR2 (red) compared to unstained control (grey) (n=3).

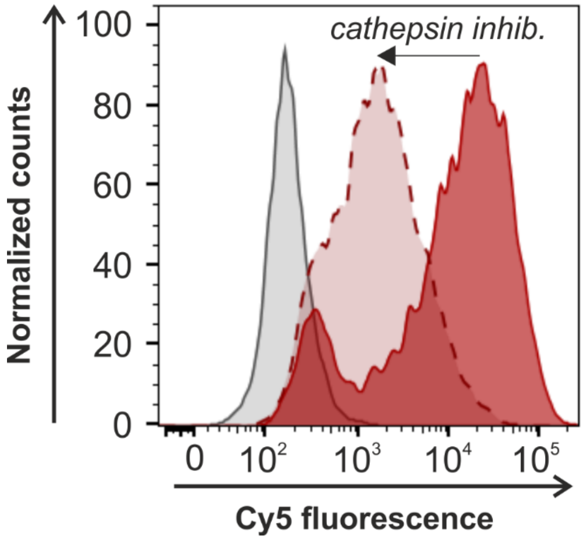

Figure S4. Cathepsin-dependent fluorescence labeling of RAW264.7 macrophages by **chemo-cat NIR**. Representative histograms after incubation of the cells with 100 nM **chemo-cat NIR** (solid line) at 37°C without any inhibitor or with pre-incubation of the cathepsin inhibitor FJD005 (5 μM) (dashed line) (n=3).

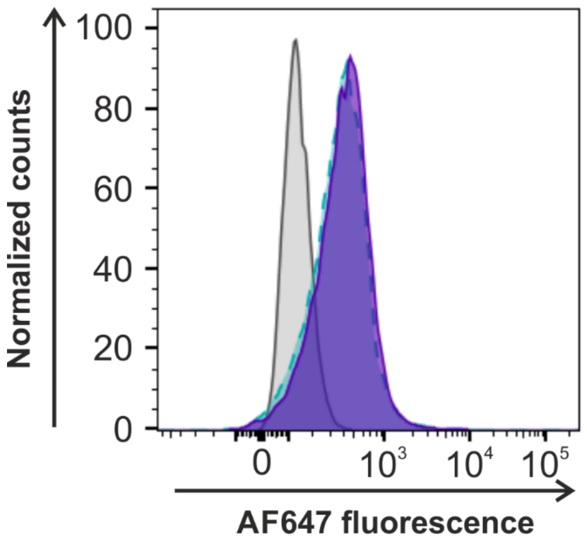

Figure S5. Cathepsin-independent fluorescence labeling of RAW264.7 macrophages by the commercial **AF647-mCCL2**. Representative histograms after incubation of the cells with 100 nM **AF647-mCCL2** (solid purple line) at 37°C without any inhibitor or with pre-incubation of the cathepsin inhibitor FJD005 (5 μM) (dashed blue line) (n=3).

Table S1. Chemical structures of FDA-approved drugs for cytotoxicity assays in RAW264.7 macrophages.

Drug	Structure	Ref
Cyclophosphamide	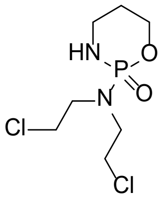	1
Fluorouracil	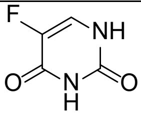	2
Cisplatin	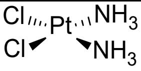	3
Docetaxel	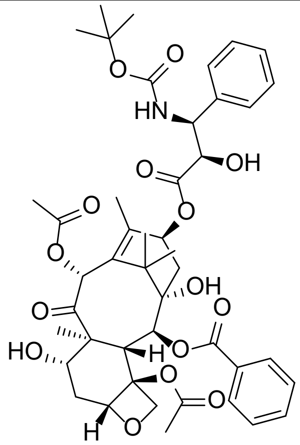	4
Bortezomib	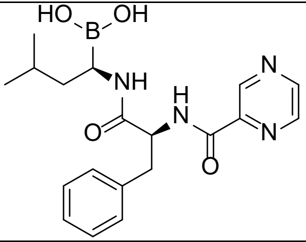	5
Gemcitabine	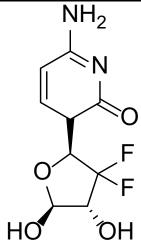	6
Doxorubicin	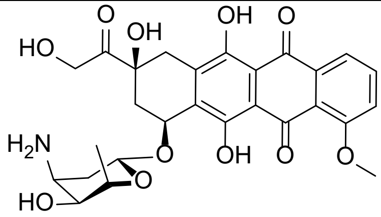	7

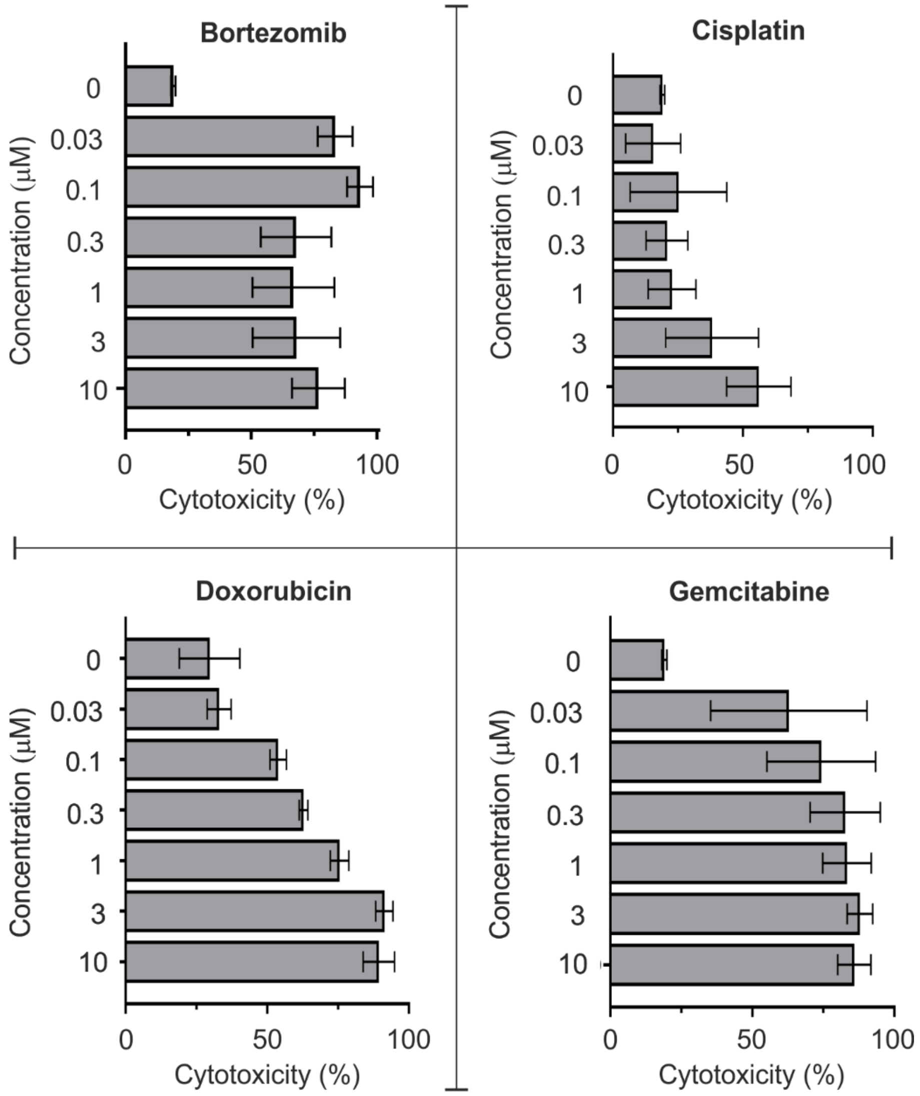

Figure S6. Dose-dependent cytotoxicity assays in RAW264.7 macrophages. Cells were incubated for 24 h with increasing concentrations of drugs as indicated. Cells were stained with AF647-Annexin V (25 nM) and propidium iodide (PI, 3 μM) before analysis by flow cytometry. Data presented as means±SD (n=3).

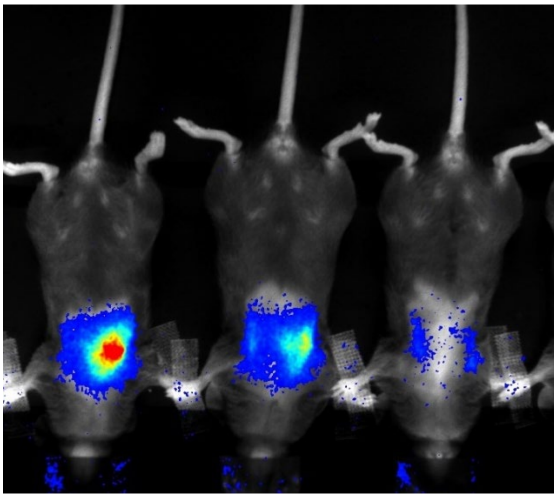

Figure S7. Bioluminescence images of syngeneic C57BL/6 mice after injection of mouse breast cancer cells (E0771-LG) via tail vein to form tumors in the lungs. The bioluminescence signals in the lungs confirm the formation of metastatic tumors. The differences in the bioluminescence signals simply correspond to the mouse-to-mouse variability in terms of tumor cell seeding and proliferation, which is inherent to the experimental lung metastasis model.

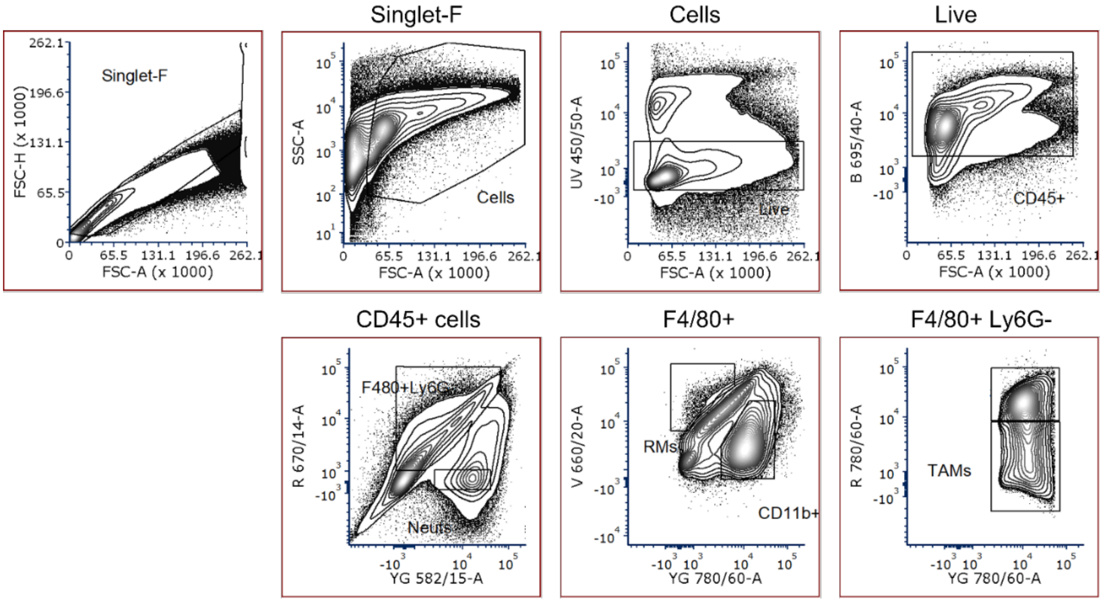

Figure S8. Gating strategy for sorting tumor-associated macrophages (TAMs) and resident macrophages (RMs) from lungs containing metastatic tumors. Markers: tumor-associated macrophages and precursor cells (TAMs): $F4/80^{+}Ly6G^{-}CD11c^{Low}CD11b^{+}$, resident macrophages (RMs): $F4/80^{+}Ly6G^{-}CD11c^{High}CD11b^{Low}$. Data were analyzed using FCS express, version 7.

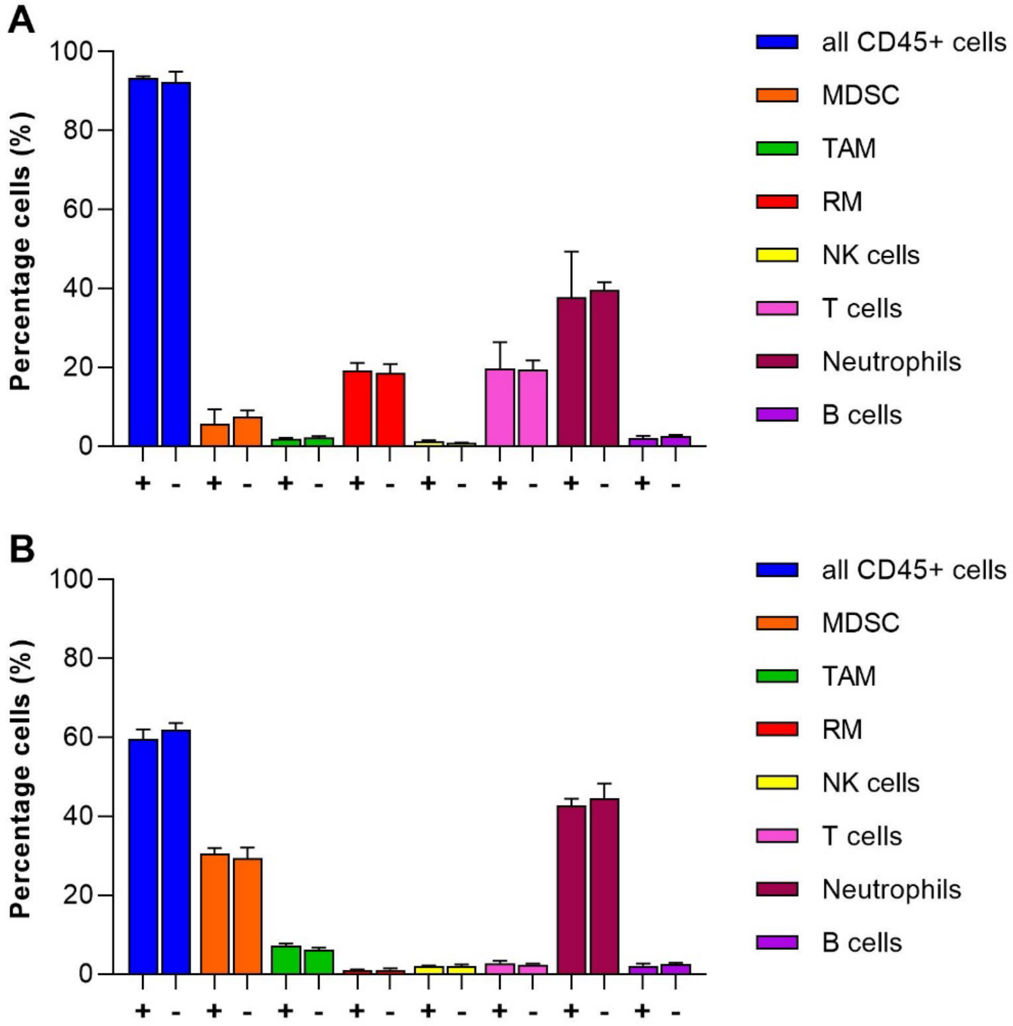

Figure S9. Immune cell composition in blood (A) and lungs (B) from mice that received *in vivo* iv administration of **chemo-cat NIR** (+) or did not receive **chemo-cat NIR** (-). Analysis was performed by flow cytometry using the gating strategy shown in Figure S11. Data presented as means±SD (n=3).

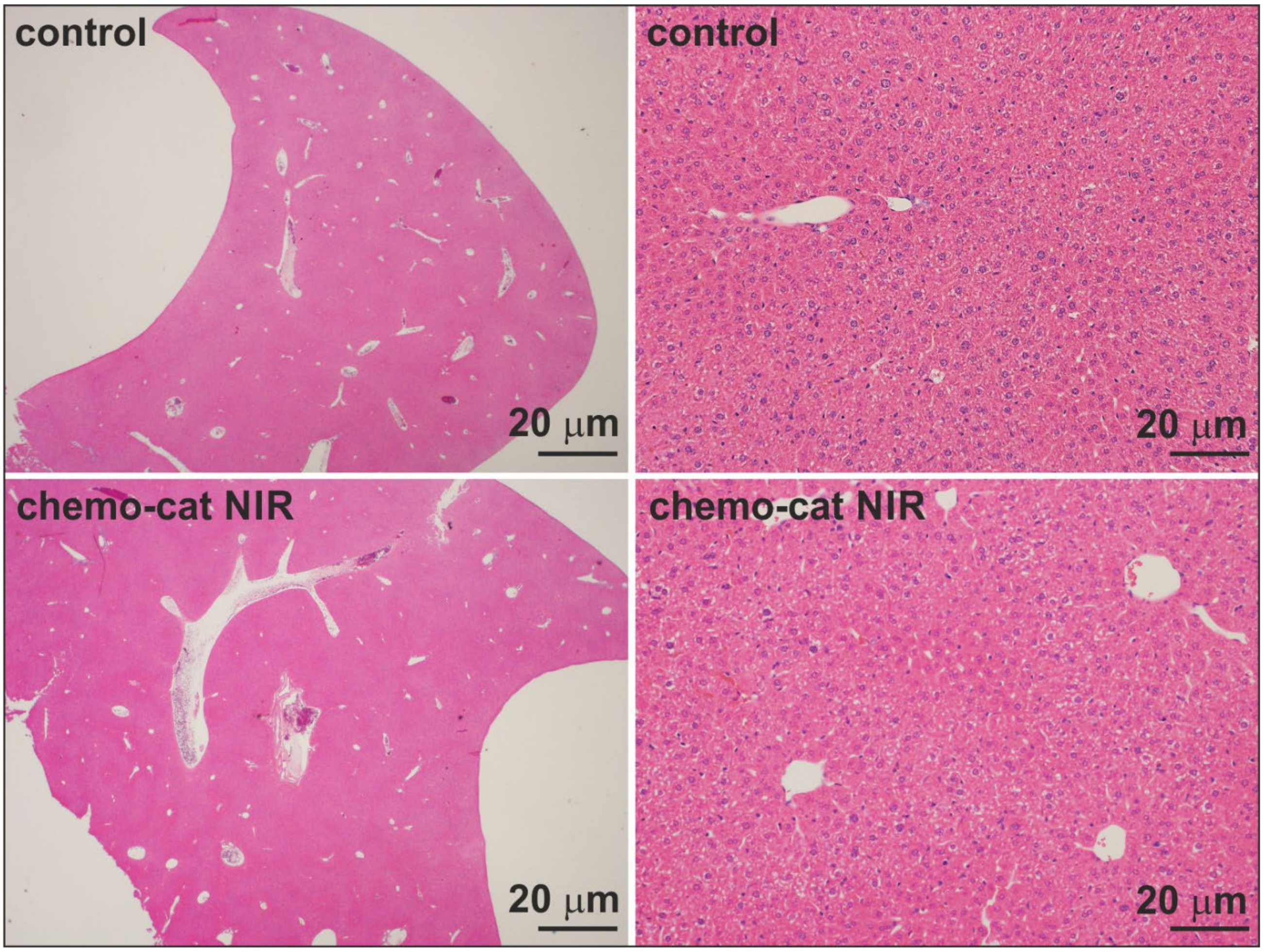

Figure S10. Histological analysis (hematoxylin and eosin staining) of harvested liver tissues from mice that did not receive **chemo-cat NIR** (top panels) or received in vivo iv administration of **chemo-cat NIR** (bottom panels). Images were taken at 2× and 20× magnification (left and right images, respectively). No pathological signs were found in mice that received **chemo-cat NIR**.

A

Target	Fluorophore	Clone	Company
CD45	PerCP/Cy5.5	30-F11	Biolegend
F4/80	FITC	Cl:A3-1	Biolegend
CD11b	BV605	M1/70	Biolegend
Ly6C	BV711	HK1.4	Biolegend
Ly6G	BV510	1A8	Biolegend
CD11c	BV650	N418	Biolegend
CD3	PE/Cy7	17A2	Biolegend
CD19	Pacific Blue	6D5	Biolegend
NK1.1	APC/Cy7	PK136	Biolegend

B

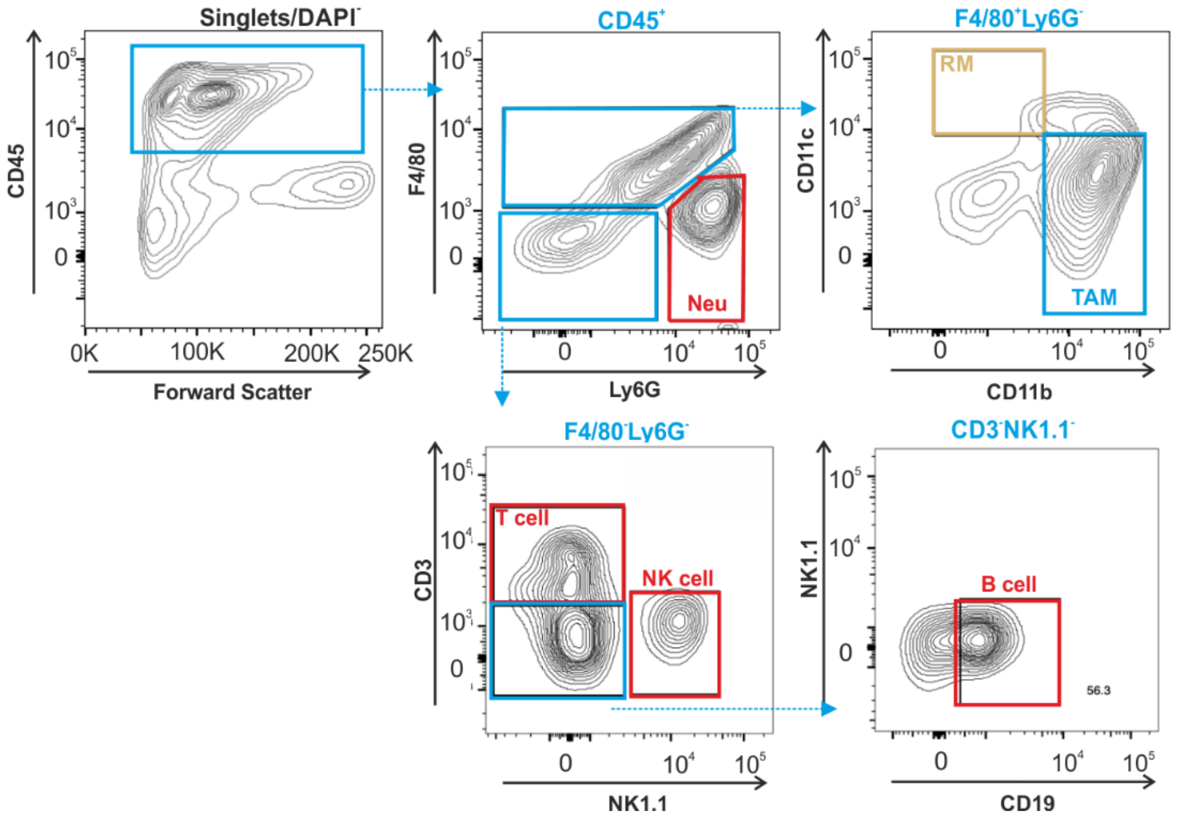

Figure S11. Antibody panel and gating strategy to identify immune cells in the lung. A) Antibodies used to identify different immune cell populations. B) Representative contour plots of digested lungs of C57BL/6 mice (day 14 after intravenous injection of E0771-LG cancer cells). Markers: Neutrophils (Neu): F4/80^{-}Ly6G^{+}, tumor-associated macrophages and precursor cells (TAMs), F4/80^{+}Ly6G^{-}CD11c^{Low}CD11b^{+}, resident macrophages (RMs): F4/80^{+}Ly6G^{-}CD11c^{High}CD11b^{Low}Ly6C^{Low}, T cells: F4/80^{-}Ly6G^{-}CD3^{+}NK1.1^{-}, NK cells; F4/80^{-}Ly6G^{-}CD3^{-}NK1.1^{+}, B cells: F4/80^{-}Ly6G^{-}CD3^{-}NK1.1^{-}CD19^{+}.

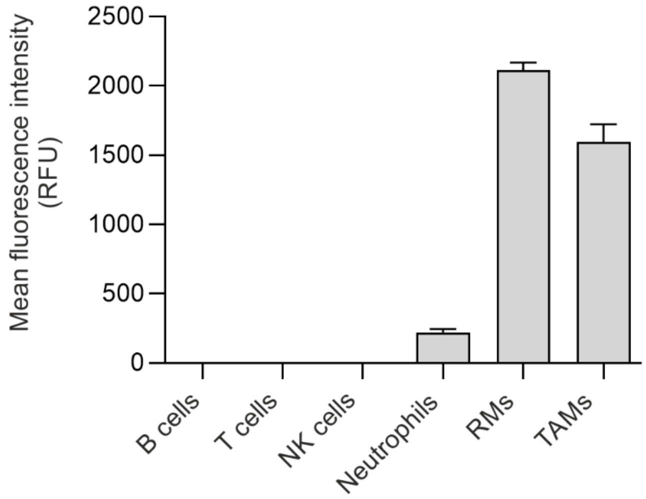

Figure S12. Geometric mean fluorescence intensities of different immune cell populations after ex vivo staining of single cell suspensions from metastatic lungs with compound **6** (500 nM) (n=3).

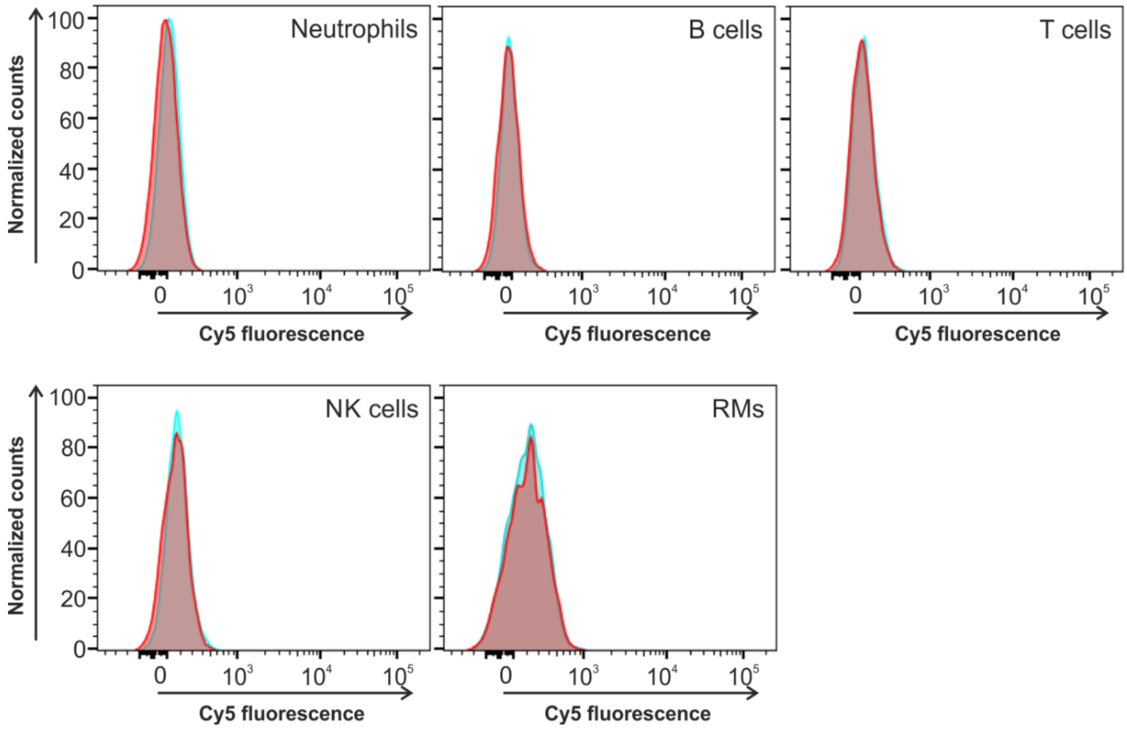

Figure S13. Flow cytometry analysis of immune cell populations labeled by **chemo-cat NIR** in vivo in tumor-bearing mice. Representative histograms of different immune cell populations (gating shown in Figure S11) after in vivo injection of **chemo-cat NIR** (0.3 nmol per mouse) where red indicates probe staining and blue indicates unstained cells (n=3).

Experimental Methods

General information. All commercial chemical reagents were used as received unless indicated otherwise. Water-sensitive reactions were performed in anhydrous solvents in flame-dried glassware under an overflow of Ar gas. Dichloromethane (DCM), diethyl ether (Et_2O), tetrahydrofuran (THF), acetonitrile (MeCN) and toluene were dried by purging over activated alumina columns in an MBraun MB SPS800 and stored over activated 3 Å molecular sieves. Technical grade n-heptane was distilled prior to use. Triethylamine (Et_3N) and N,N-diisopropylethylamine (DIPEA) were distilled over calcium hydride and stored over potassium hydroxide pellets. Reactions were monitored using analytic thin-layer chromatography (TLC) on glass TLC plates (60G, F_{254}, Merck) with UV detection at 254 nm and by staining with either a solution of Baeyer's reagent ($KMnO_4$ (10 g L^{-1}) and K_2CO_3 (50 g L^{-1}) in water) or by staining with a ninhydrin-solution (10 g L^{-1}) in BuOH/AcOH (99/1; v/v) and subsequent charring at ~150 °C. Flash column chromatography was carried out on SiliaFlash® silica gel (40–63 µm, 60 Å, SiliCycle). Analytical LCMS was performed using a hybrid Thermo Finnigan/Shimadzu system equipped with a Phenomenex Gemini NX C18 column (3 µm, 110 Å, 50 mm x 2.0 mm) using mobile phases A: 1% aq. HCOOH, B: 1% HCOOH in MeCN, with detection at 210–600 nm by a diode array detector coupled to a LCQ fleet mass spectrometer (Thermo Finnigan). Preparative HPLC was performed using a Shimadzu system equipped with a Phenomenex Gemini NX-C18 column (5 µm, 110 Å, 150 mm x 21.2 mm) in combination with mobile phases A: 0.1% TFA in H_2O, B: 0.1% TFA in MeCN, with detection at 214/254 nm. Electrospray ionisation (ESI) mass spectrometry (MS) was performed on a LCQ Advantage Max (Thermo Finnigan). High resolution mass spectrometry (HRMS) (ESI-TOF) was recorded on a AccuTOF CS JMS-T100CS mass spectrometer (JEOL), equipped with an electrospray ion source in positive mode. ^{1}H NMR and ^{13}C NMR spectra were recorded on a Brüker Avance III 400 (9.4 T) or Brüker Avance III 500 (11.7 T) spectrometer. Chemical shifts (δ) are reported in ppm relative to tetramethylsilane as internal standard or the residual signal of the deuterated

solvent was used as reference point. Coupling constants (J) are given in Hz. All ^{13}C APT experiments were proton decoupled. Spectroscopic data was recorded on Cytation 3 (Biotek).

Chemical synthesis

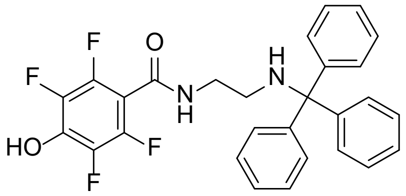

2,3,5,6-tetrafluoro-4-hydroxy-N-(2-(tritylamino)ethyl)benzamide (2). Trt-EDA-AcOH (1.81 g, 5 mmol, 1 eq) was taken up in DCM (500 mL) and washed with sat. $NaHCO_3$, sat. NaCl, dried over Na_2SO_4, filtered and concentrated. The oily residue was taken up in dry DCM/DMF (4:1, 25 mL) and to this was added 2,3,5,6 tetrafluoro-4-hydroxy benzoic acid (946 mg, 4.5 mmol, 0.9 eq), EDCI (854 mg, 5.5 mmol, 1.1 eq), HOBt (842 mg, 5.5 mmol, 1.1 eq) and N-methyl morpholine (0.6 ml, 5.5 mmol, 1.1 eq). The mixture was stirred for 16 h at r.t., diluted with water and extracted with EtOAc (x3). The extracts were washed with sat. NaCl, dried over Na_2SO_4, filtered and concentrated. Column chromatography (10 to 40% EtOAc in PetEt) yielded the title compound as white solid (1.97 g, 3.98 mmol, 80%). R_f = 0.5 (5% MeOH in DCM); ^1H NMR (500 MHz, Acetone) δ 7.99 (t, *J* = 6.1 Hz, 1H), 7.53 (d, *J* = 7.3 Hz, 5H), 7.30 (t, *J* = 7.7 Hz, 6H), 7.20 (t, *J* = 7.3 Hz, 2H), 3.60 (q, *J* = 6.1 Hz, 2H), 2.38 (t, *J* = 6.1 Hz, 2H); ^{13}C NMR (126 MHz, Acetone) δ 158.1, 146.3, 144.9, 143.0, 138.9, 137.1, 128.6, 127.7, 126.2, 107.1, 105.0, 70.6, 43.5, 40.3.

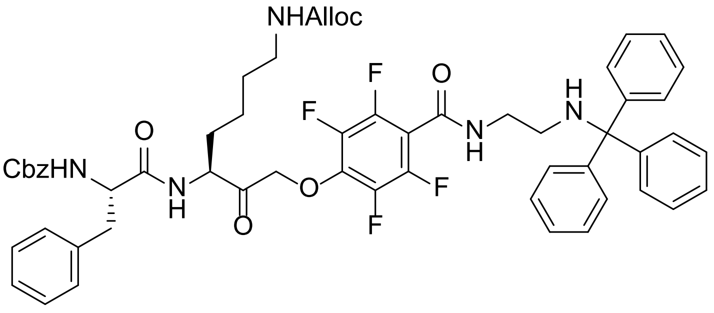

Z-FK(Alloc)-PMK-EDA-NHTrt (3). Z-FK(Alloc)-CMK (109 mg, 0.2 mmol) was taken up in dry DMF (1 mL) and was treated with **2** (109 mg, 0.22 mmol, 1.1 eq) and potassium fluoride (17.4

mg, 0.3 mmol, 1.5 eq) for 16 h at 60 °C. The reaction was diluted with water, extracted with EtOAc, and the extracts were washed with sat. NaCl, dried over Na_2SO_4, filtered and concentrated. Column chromatography (0 to 10% acetone in DCM) yielded white powder. The intermediate was directly used in the next reaction without further purification or analysis.

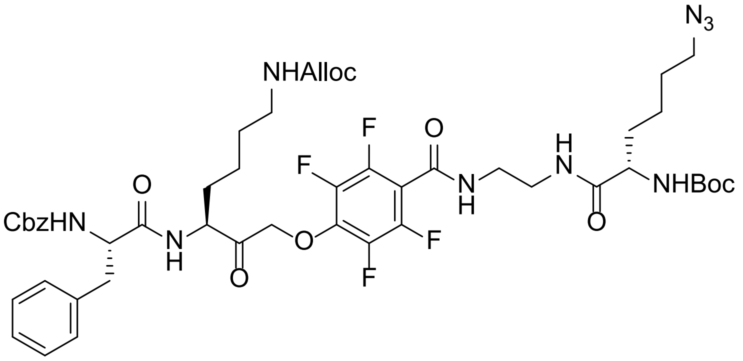

Z-FK(Alloc)-PMK-EDA-K(N_3)-NHBoc (4). The intermediate **3** was treated with TFA/TIS/DCM (2/2/96, 1 mL) for 1 h at r.t. and the mixture was co-evaporated with toluene (x3). The crude was taken up in dry DMF (1 mL) and was treated with Boc-L-Lys(azido)-OSu (81.4 mg, 0.24 mmol, 1.2 eq) and DIPEA (104 µl, 0.6 mmol, 3 eq) for 16 h at r.t., before pouring into 0.1 N HCl and extracting with EtOAc (x3). The extracts were washed with sat NaCl, dried over Na_2SO_4, filtered and concentrated. Column chromatography (10 to 40% acetone in DCM) yielded the title compound as white solid (190 mg, 0.187 mmol, 94%). R_f = 0.7 (40% acetone in DCM); ^{1}H NMR (500 MHz, DMSO) δ 8.82 (t, *J* = 5.5 Hz, 1H), 8.50 (d, *J* = 7.4 Hz, 1H), 7.94 (t, *J* = 5.6 Hz, 1H), 7.63 (d, *J* = 8.2 Hz, 1H), 7.36 – 7.24 (m, 10H), 7.21 – 7.14 (m, 2H), 6.80 (d, *J* = 8.1 Hz, 1H), 5.89 (ddt, *J* = 17.3, 10.6, 5.4 Hz, 1H), 5.25 (dq, *J* = 17.2, 1.8 Hz, 1H), 5.19 – 5.11 (m, 2H), 5.03 (d, *J* = 17.9 Hz, 1H), 4.97 (s, 2H), 4.44 (d, *J* = 5.5 Hz, 2H), 4.35 – 4.25 (m, 2H), 3.87 (td, *J* = 8.5, 4.9 Hz, 1H), 3.32 – 3.23 (m, 6H), 3.22 – 3.13 (m, 1H), 3.04 – 2.90 (m, 3H), 2.80 (dd, *J* = 13.8, 9.9 Hz, 1H), 1.79 – 1.70 (m, 1H), 1.67 – 1.58 (m, 1H), 1.57 – 1.44 (m, 4H), 1.37 (s, 11H), 1.34 – 1.26 (m, 4H); ^{13}C NMR (126 MHz, DMSO) δ 203.9, 172.8, 172.6, 157.8, 156.4, 155.8, 144.6, 142.6, 140.9, 138.9, 138.3, 137.6, 137.4, 134.3, 129.7, 128.7, 128.5, 128.2, 128.0, 126.8, 117.3,

110.6, 78.4, 75.3, 65.8, 64.6, 56.5, 56.0, 54.6, 51.0, 38.4, 37.6, 31.9, 29.5, 29.2, 28.6, 28.4, 23.1, 22.8.

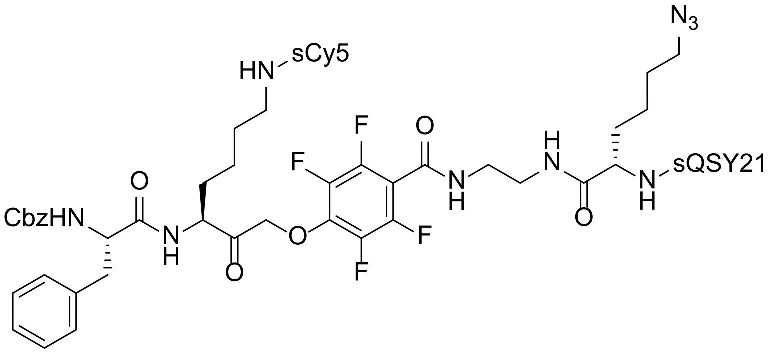

Z-FK(sCy5)-PMK-EDA-K(N_3)-NH-sQSY21 (5). Intermediate **3** (190 mg, 0.187 mmol) was taken up in THF and bubbled with argon for 10 min, before adding palladium tetrakis triphenylphosphine (22 mg, 0.02 mmol, 0.1 eq) and 1,3-dimethylbarbituric acid (88 mg, 0.56 mmol, 3 eq). The reaction was stirred for 10 min at r.t. before diluting with MeOH and subsequent purification by preparative HPLC (5 to 60% MeCN in water, 12/20 min). Lyophilisation (x2) yielded white powder (159 mg, 0.171 mmol, 91%). The refined intermediate (27 mg, 30 µmol, 1.1 eq) was taken up in dry DMSO (1 ml) and treated with Cy5-OSu (20 mg, 27 µmol, 1 eq) and DIPEA (14 µl, 81 µmol, 3 eq) for 4 at r.t. in the dark. The reaction was diluted with MeCN, separated with preparative HPLC and subsequent lyophilisation (x2) yielded blue powder (28 mg, 18 µmol, 67%). Purity >95% as determined with HPLC. No further analysis was performed, and half of the intermediate was directly used in the next reaction. The intermediate (15 mg, 9.6 µmol, 1 eq) was treated with TFA/DCM (1:1, v:v, 1 mL) for 30 min at r.t. and was co-evaporated with toluene (x2). The crude residue was taken up in dry DMSO and QSY21-OSu (9.1 mg, 9.6 µmol, 1 eq) and DIPEA (5.2 µL, 29 µmol, 3 eq) were added. The reaction was stirred for 16 h at r.t. in the dark. The mixture was diluted with MeCN, followed by HPLC purification (5 to 65% MeCN in H_2O, 28/30 min) and lyophilisation (x2) to yield the title compound as blue solid (8 mg, 3.5 µmol, 37%).

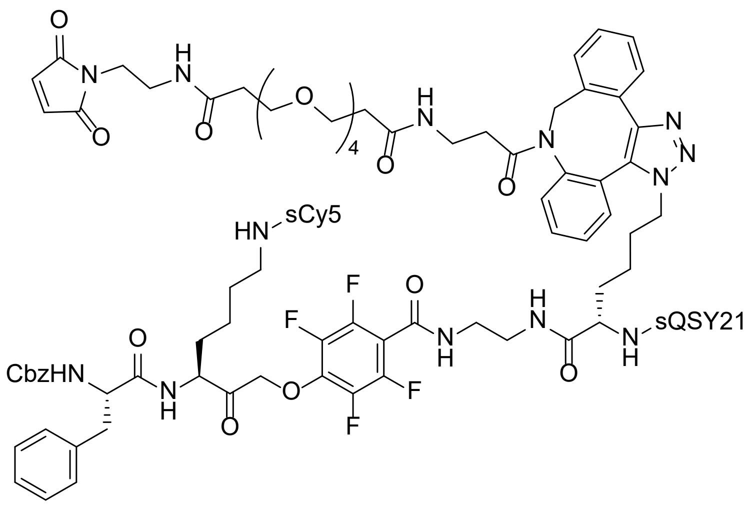

Z-FK(sCy5)-PMK-EDA-K(PEG4-Mal)-NHsQSY21 (5b). Azide **5** (4 mg, 1.76 µmol) was taken up in dry DMSO (400 µL) and a solution of DBCO-PEG4-Mal (2.5 mg, 3.7 µmol, 2.1 eq) in dry DMSO (100 µL) was added. The reaction was stirred for 16 h at r.t. in the dark. The mixture was diluted with MeOH and directly applied to HPLC purification (5 to 60% MeCN in H_2O, 28/30 min). Subsequent lyophilisation yielded the final product as blue powder (2.8 mg, 0.95 µmol, 54%). Purity >99% as determined with HPLC (5 to 95% MeCN in H_2O, 25/35 min); ESI-MS m/z calc. for $C_{148}H_{158}F_4N_{18}O_{33}S_5$ $[M+2H]^{2+}$ = 1477.0, found 1477.1.

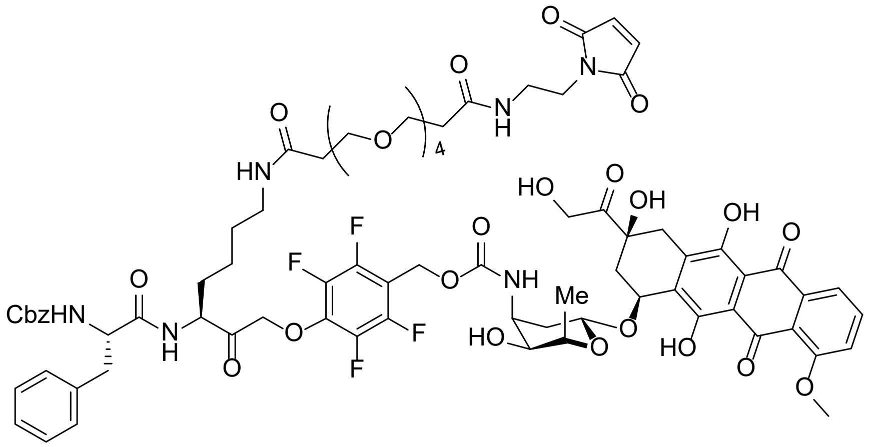

Z-FK(PEG4-Mal)-PMK-Dox (8). Z-FK(NH_2)-PMK-Dox **7**[8] (5.3 mg, 4.07 µmol) was taken up in dry DMSO (1 mL) and OSu-PEG4-Mal (2.5 mg, 4.86, 1.2 eq) and DIPEA (1.6 µL, 9.2 µmol, 3 eq) were added. The reaction was stirred for 2 h, before diluting with MeOH (1.5 mL) followed by HPLC purification (20 to 70% MeCN in H_2O, 38/45 min). Subsequent lyophilisation (x2)

yielded red powder (5.2 mg, 3.28 µmol, 81%). ^{1}H NMR (500 MHz, DMSO) δ 14.04 (s, 0H), 13.29 (s, 0H), 8.49 – 8.43 (m, 1H), 8.00 (t, *J* = 5.7 Hz, 1H), 7.95 – 7.88 (m, 2H), 7.77 (q, *J* = 5.9 Hz, 1H), 7.68 – 7.59 (m, 2H), 7.35 – 7.22 (m, 10H), 7.22 – 7.12 (m, 2H), 7.00 (s, 2H), 6.97 (dd, *J* = 7.7, 3.2 Hz, 1H), 5.45 (s, 1H), 5.27 – 5.16 (m, 2H), 5.15 – 4.98 (m, 3H), 4.95 (s, 2H), 4.92 (s, 1H), 4.67 (s, 1H), 4.58 (s, 2H), 4.33 – 4.24 (m, 2H), 4.24 – 4.18 (m, 1H), 4.15 (q, *J* = 6.5 Hz, 1H), 3.99 (s, 3H), 3.74 – 3.65 (m, 1H), 3.62 – 3.55 (m, 4H), 3.49 (s, 8H), 3.48 – 3.43 (m, 6H), 3.34 (s, 17H), 3.15 (q, *J* = 5.8 Hz, 2H), 3.04 – 2.88 (m, 6H), 2.84 – 2.73 (m, 1H), 2.33 (t, *J* = 7.3 Hz, 2H), 2.28 (q, *J* = 6.2 Hz, 2H), 2.23 – 2.16 (m, 1H), 2.15 – 2.09 (m, 1H), 1.83 (td, *J* = 13.0, 3.9 Hz, 1H), 1.77 – 1.68 (m, 1H), 1.68 – 1.59 (m, 1H), 1.53 – 1.41 (m, 2H), 1.40 – 1.17 (m, 4H), 1.12 (d, *J* = 6.4 Hz, 4H); ^{13}C NMR (126 MHz, DMSO) δ 214.2, 204.3, 186.9, 172.5, 171.2, 170.3, 169.9, 161.3, 156.6, 155.0, 146.5, 144.5, 141.0, 138.2, 138.1, 137.3, 136.7, 136.0, 135.2, 135.0, 134.6, 129.6, 128.7, 128.5, 128.2, 128.0, 126.8, 120.5, 120.2, 119.5, 111.3, 111.1, 100.7, 75.4, 70.2, 70.2, 70.2, 70.1, 70.0, 70.0, 69.4, 68.3, 67.3, 67.1, 65.7, 64.1, 57.1, 53.3, 47.8, 38.9, 38.6, 37.6, 37.1, 36.6, 34.5, 34.4, 32.6, 30.1, 29.2, 22.9, 17.5; Purity > 95% as determined with LCMS (5 to 95% MeCN in H_2O, 7.6/10 min); ESI-MS m/z calc. for $C_{77}H_{86}F_4N_6O_{26}$ [M+Na$^+$] = 1609.5 found 1609.6; HRMS (ESI+) m/z calc. for $C_{77}H_{86}F_4N_6O_{26}$ [M+H$^+$] = 1609.5426 found 1609.5469.

mCCL2 conjugates. 100 µL of the compounds **5b** or **8** (1 mM) in DMSO:buffer (1:4) were added to mCCL2-thiol (200 µg, 0.022 µmol) in phosphate buffer (total volume: 200 µL, pH 6.5). The mixtures were stirred a r.t. for 2 h in the dark, and the crudes were purified by reverse phase HPLC [PhenylHexyl column, eluents: H_2O-MeCN (0.1% HCOOH)] to obtain the **chemo-cat NIR** or **chemo-cat DOX** as blue and orange solids respectively (20-30% yield, determined by UV absorption). Purities >90% as determined with HPLC at 650 nm (5 to 95% MeCN in H_2O, 9.8/12 min). ESI-MS m/z calc. [M+H$^+$] = 11,837.8 found 11,837.0.

Cell culture. RAW264.7 cells were obtained from American Type Culture Collection (ATCC). The cells were cultured in DMEM containing high glucose, stable glutamine (GlutaMAX™), sodium pyruvate and phenol red, which was supplemented with 10% foetal calf serum (FCS) and antibiotics (100 U mL^{-1} penicillin, 100 µg mL^{-1} streptomycin, and 250 ng mL^{-1} amphotericin B). Cells were maintained in a humidified atmosphere at 37°C with 5% CO_2 and harvested at 80-90% confluence. E0771 mouse mammary adenocarcinoma cells were provided by Dr. E. Mihich (Roswell Park Cancer Institute, NY), previously reported selection processes were carried out to generate highly metastatic derivative cell line E0771-LG.[9]

In vitro labeling and SDS-PAGE. RAW macrophages (~10^5 cells in 50 µL complete medium) were incubated with 2.5 µM cathepsin inhibitor FJD005[8] (100x in DMSO) or 2.5 µM CCR2 antagonist RS504393 (100x in DMSO) or vehicle for 5 min at 37 °C, followed by labeling with 250 nM compound **6** (100x in DMSO) or 250 nM **chemo-cat NIR** (100x in MQ) for the indicated times at 37 °C. The cells were centrifuged at 10,000 *g* for 1 min at r.t., the supernatants were removed, and the cells were taken up in 9 µL hypotonic lysis buffer (50 mM PIPES pH 7.4, 10 mM KCl, 5 mM $MgCl_2$, 4 mM DTT, 2 mM EDTA, and 1% NP40). The lysates were incubated on ice for 5 min, followed by centrifugation at 21,130*g* for 15 min at 4 °C. The cleared lysates were diluted with 3 µL Laemmli's 4x sample buffer (40% glycerol, Tris/HCl (0.2 M, pH 6.8), 8% SDS, 10% BME, and 0.04% bromophenol blue) and the mixtures were denatured over 5 min at 95 °C. The samples were spun down and separated by SDS PAGE (15%, 15 min at 80 V, 1.5-2 h at 120 V). The gels were analyzed by in-gel fluorescence scanning on a Typhoon Amersham flat-bed laser scanner (GE Healthcare) and equal protein loading was confirmed by staining with Coomassie® Brilliant Blue R-250 (Schmidt GmbH).

Live-cell fluorescence microscopy and confocal microscopy. RAW264.7 macrophages (~7.5×10^4 in 200 µL phenol red-free, complete medium) were seeded in a 1 cm^2 glass-bottom

and cultured overnight in a 5% CO_2 incubator at 37 °C. The cells were treated with 2.5 µM cathepsin inhibitor FJD005 (100x in DMSO) or 2.5 µM CCR2 antagonist RS504393 (100x in DMSO) or vehicle for 5 min at 37 °C, followed by labeling with 250 nM compound **6** (100x in DMSO) or 250 nM **chemo-cat NIR** (100x in MQ) at 37 °C. Representative images were taken (~5 min imaging) and the samples were returned to the incubator. The cells were imaged with a DMI 6000B microscope (Leica) using an HC PL APO 1.40-0.60 mm oil-immersion lens. After the last time point, the cells were washed with PBS, fixed with 4% PFA in PBS for 20 min at r.t., washed with PBS (x2) and the samples were permeabilized with 0.5% saponin in PBS for 20 min at 4 °C. Cells were blocked with 3% BSA, 1% filtered FCS, 0.5% saponin in PBS for 30 min at 4 °C and cells were incubated with rabbit anti-LAMP1 in 1% BSA, 0.5% saponin in PBS for 20 min at 4 °C. This was followed by staining with anti-rabbit AF488 in 1% BSA, 0.5% saponin in PBS for 20 min at 4 °C. The cells were washed with PBS and stored in 0.5% PFA in PBS at 4 °C in the dark. Finally, the cells were incubated with 1 µg mL^{-1} DAPI (4′,6-diamidino-2-phenylindole) for 30 min in PBS, followed by washing with PBS (2x). The samples were imaged on a SP8 confocal microscope (Leica) with an HC PL APO CS2 63x/1.20 water objective. Image analysis was performed using FIJI software.

Transwell assays. RAW264.7 cells were passaged one day prior to the experiment to achieve a confluency of 80-85% for the assay. Cell media was replaced with pre-warmed PBS (-Ca^{2+}, -Mg^{2+}) and cells were gently lifted using a cell scrapper in PBS. Cells were resuspended at $1.5x10^6$ in RPMI and 100 µL were added to transwell inserts with 5 µm membrane. The inserts were placed into 24-well plates in RPMI or RPMI containing 10 ng mL^{-1}, 30 ng mL^{-1} or 100 ng mL^{-1} mCCL2 (Preprotech) or **chemo-cat NIR** cells were allowed to migrate for 2 h in a humidified atmosphere at 37°C with 5% CO_2. The wells were transferred into a new plate containing PBS and washed thrice with PBS. To prevent dehydration of cells, PBS was kept within the transwell. Non-migrated cells were gently removed using cotton buds and wells rinsed once more with

PBS. Cells were fixed with ice cold acetone;MeOH (1:1) for 5 min at r.t. and washed twice with PBS. Cell nuclei were stained with 3 µM Hoechst 33342 (Invitrogen) in PBS, 1% BSA for 20 min at r.t. Membranes were cut using a scalpel, placed on microscope slide with cells facing up and mounted using Prolong Gold. Cells were imaged on Leica SP8 confocal ($\lambda_{exc.}$: 405 nm, $\lambda_{em.}$: 450 nm). Migration experiments were performed in triplicates, at least three field of views per replicate were used and migrated cell numbers determined using ImageJ.

Flow cytometry. Drug screening. To select the most potent chemotherapeutic to couple to the cathepsin-activated linker and mCCL2, the ability of diverse drugs to induce apoptosis and necrosis in RAW264.7 cells were screened. Briefly, cells were detached as described above and resuspended at 4×10^5 cells mL^{-1} in RPMI, 50 µL/well were transferred into BD 96 well flat bottom plate. After adjusting cell media to 150 µL/well, cells were allowed to adhere and proliferate for 18 h in a humidified atmosphere at 37°C with 5% CO_2. Drugs were dissolved at 10 mM, diluted to the indicated working concentrations and incubated with the cells for 24 h. After treatment, cells were centrifuged (300 *g*, 5 min, r.t.), washed once with PBS before addition of 0.05% Trypsin, 0.02% ethylenediaminetetraacetic acid (EDTA) in Hank's balanced salt solution (HBSS) and incubation at 37°C for 5 min. Detached cells were combined in 96-well round bottom plate followed by Pacific Blue-Annexin V and propidium iodide (PI) staining. Briefly, 25 nM Pacific Blue-Annexin B was added in HEPES-NaCl containing 0.1 % BSA and 2 mM $CaCl_2$ for 20 min at r.t. and 3 µM propidium iodide was added directly before analysis. Fluorescence emission was acquired on the Attune Nxt under the following excitation/emission filters: Pacific Blue-Annexin V (405/450 nm), PI (561/580 nm). Data was analyzed using the FlowJo X software.

In vitro experiments in RAW264.7 macrophages. Macrophages were detached, centrifuged (300 *g*, 5 min, r.t.) and 10^5 cells per staining were used. Cells along appropriate controls were

incubated with indicated concentrations of **chemo-cat NIR**, **mCCL2-AF647** or compound **6** in HEPES-NaCl containing 0.1 % BSA for 1 h at 37°C. 1 µM cathepsin inhibitor FJD005 was added 1 h at 37°C prior to staining. Additional antibody staining included anti-CCR2-AF647 (1:40), anti-CCR2-FITC (1:40) and were performed for 20 min at 4°C in HEPES-NaCl containing 0.1 % BSA. Fluorescence was acquired on 5L LSR Fortessa II using Diva software under the following excitation/emission filters: **chemo-cat NIR**, **mCCL2-AF647**, compound **6** or anti-CCR2-AF647 (647/680 nm), anti-CCR2-FITC (488/525 nm).

In vivo staining of mouse metastatic lungs. All animal work was conducted under the control of the UK Home Office at the University of Edinburgh and by the University of Edinburgh animal welfare committee (PPL: P9C3F7964). C57BL/6 mice with metastatic lung tumours (day 14 after tail vein injection of EO771-LG cells) were injected with 5 µM of **chemo-cat NIR** or compound **6**. After 2 h, lungs were isolated, digested using an enzyme cocktail (Lung Dissociation Kit, Miltenyi Biotec) for 30 min at 37°C and red blood cells lysed (5 min, RBC lysis buffer). 5 mL FACS buffer was added and filtrated with a 40 µm cell strainer. Cells were centrifuged twice at 300 *g*, 5 min, 4°C in 5 mL FACS buffer and again filtrated with a 40 µm cell strainer. 5×10^5 cells per staining were transferred into flow tubes and FcR block for 15 min at 4°C. To identify specific immune cell populations, the antibodies detailed in Figure S9 were used. Cells were incubated for 30 min, 4°C, centrifuged (300 *g*, 5 min, 4°C) and re-suspended in 300 µL FACS-buffer containing 3 µg/mL DAPI for acquisition on 6L LSR Fortessa II under the following excitation/emission filters: PerCP/Cy5.5-CD45 (488/710), FITC-F4/80 (488/525), BV605-CD11b (405/610), BV711-Ly6c (405/710), BV510-Ly6G (405/525), BV650-CD11c (405/660), PE/Cy7-CD3 (651/780), Pacific Blue-CD19 (405/450), APC/Cy7-NK1.1. (647/780), BMV109 and mCCL2-FJD305 (647/670). Data was analyzed using FlowJo X software.

Ex vivo staining of mouse metastatic lungs. Metastatic lungs were lysed as described above. 5x10^5 cells were incubated with **chemo-cat NIR** or compound **6** (all at 500 nM) for 1 h, 37°C in aMEM supplemented with 10% FBS and mixed gently every 20 min. After 1 h, cells were centrifuged (300 *g*, 5 min, 4°C) and FcR block and staining performed as described above. Fluorescence emission was acquired on 6L LSR Fortessa II and analyzed using FlowJo X software.

Cell viability of prodrugs vs doxorubicin. To compare the efficacy of prodrugs vs doxorubicin, we used the TACSR MTT Cell Proliferation assay (Trevigen). Briefly, cells were detached as described above and resuspended at 1.5×10^5 cells mL^{-1} in RPMI, 100 µL/well were transferred into BD 96-well flat bottom plates. After adjusting cell media to 150 µL/well, cells were allowed to adhere and proliferate for 12 h in a humidified atmosphere at 37°C with 5% CO_2. Prodrugs and doxorubicin were diluted to the indicated working concentrations and incubated with the cells for 24–48 h. The cell media was removed, and fresh media was added, and the MTT Cell Proliferation assay was carried out according to manufacturer's instructions and absorbance was acquired at 570 nm on the Synergy HT spectrophotometer (Biotek). Cell viabilities were normalized to untreated cells.

Statistical analysis. Statistical differences were analyzed two-sided unpaired *t* tests using Graphpad software. Independent experiments were performed at least three times, unless otherwise mentioned.

NMR Spectra

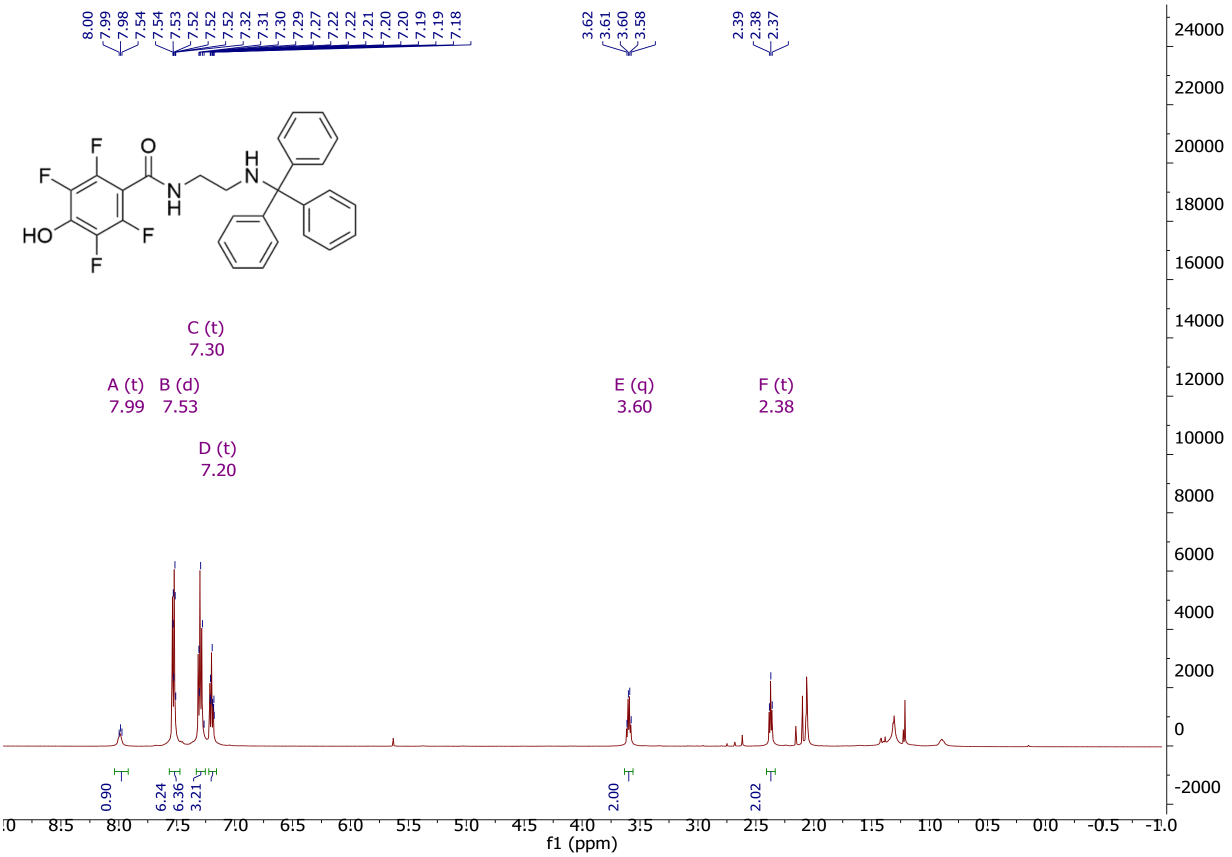

C (t)
7.30
A (t)
7.99
B (d)
7.53
E (q)
3.60
F (t)
2.38
D (t)
7.20
f1 (ppm)

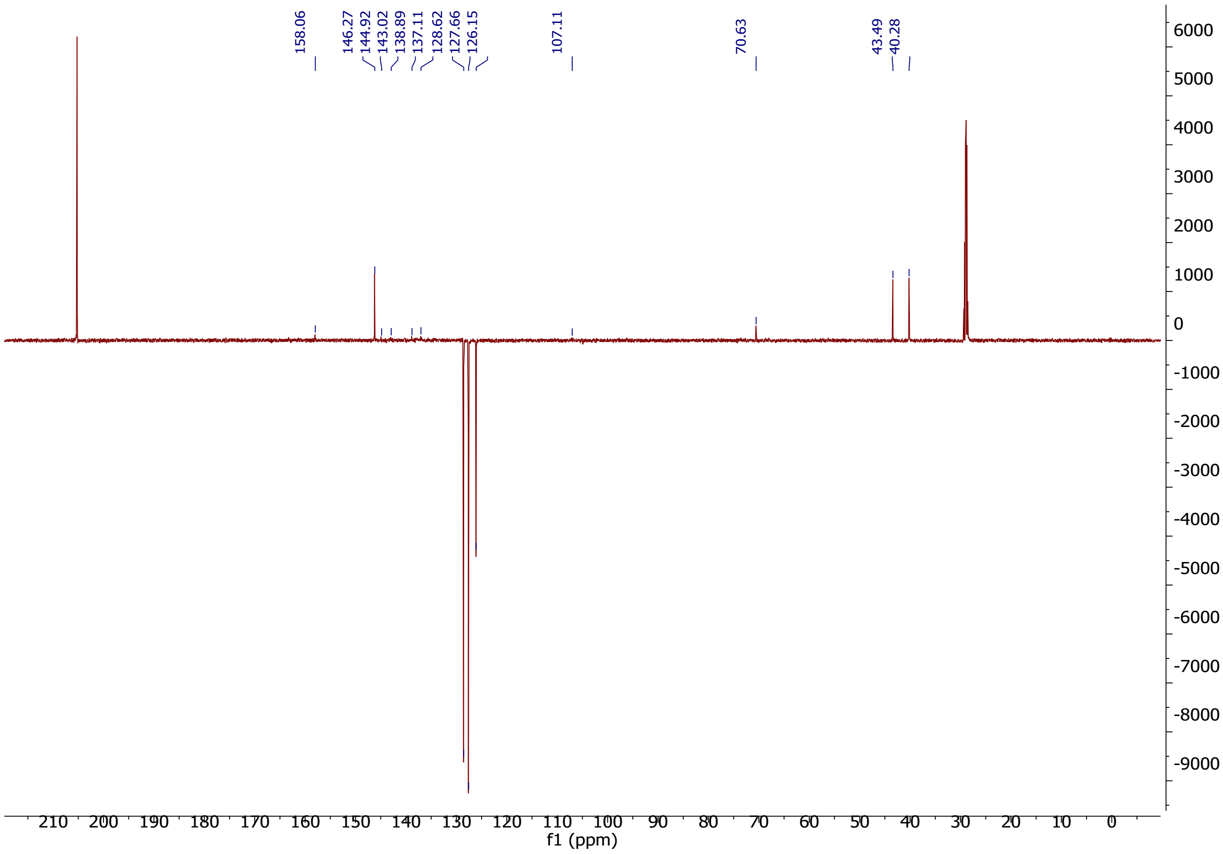

158.06
146.27
144.92
143.02
138.89
137.11
128.62
127.66
126.15
107.11
70.63
43.49
40.28
f1 (ppm)

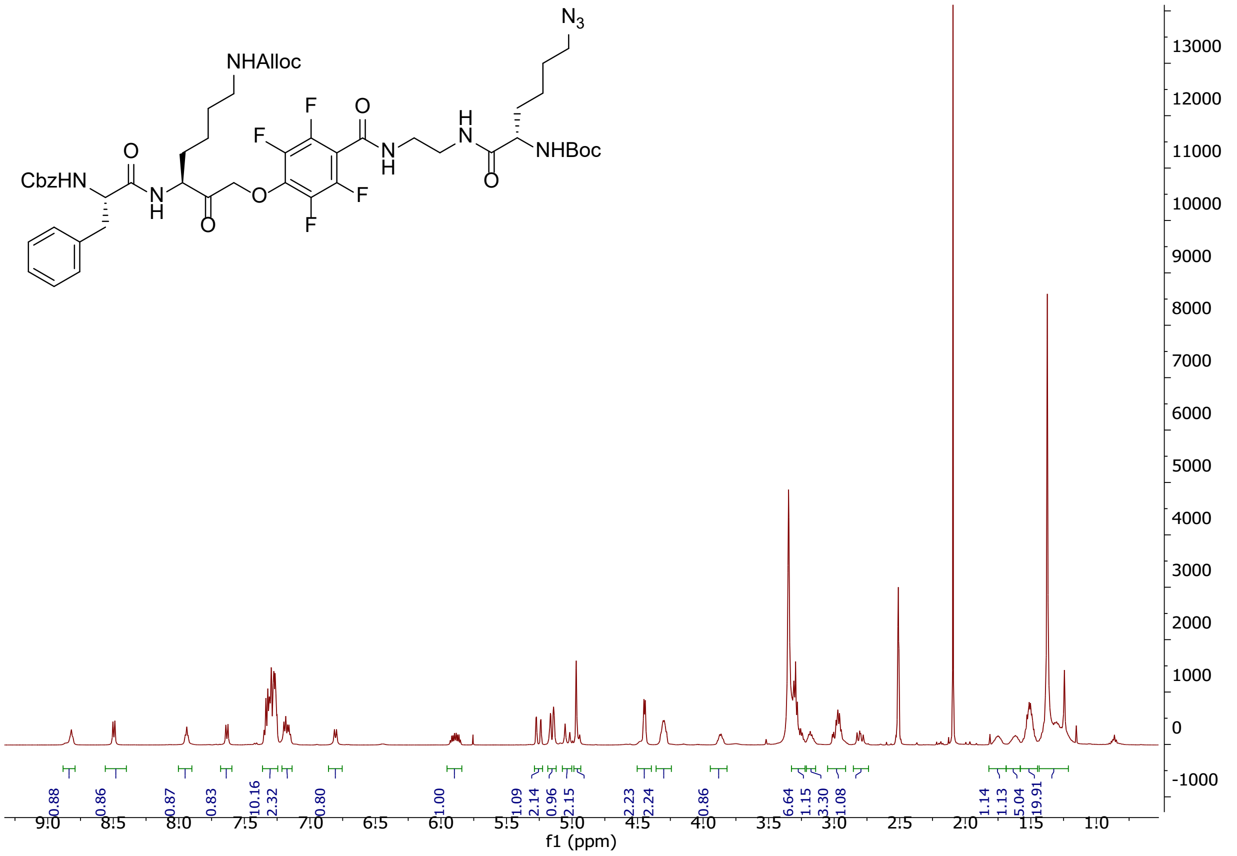
NHAlloc
N3
CbzHN
NHBoc
f1 (ppm)

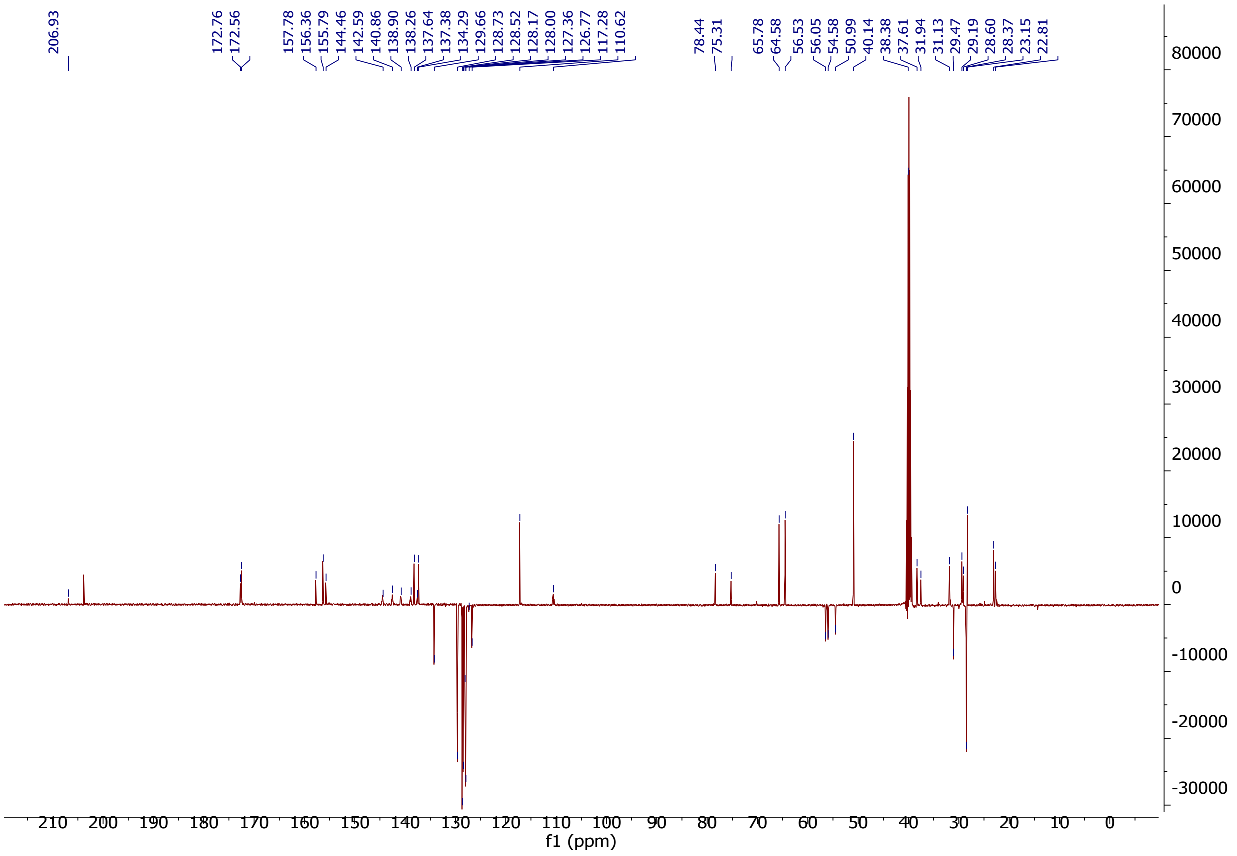
206.93
172.76
172.56
157.78
156.36
155.79
144.46
142.59
140.86
138.90
138.26
137.64
137.38
134.29
129.66
128.73
128.52
128.17
128.00
127.36
126.77
117.28
110.62
78.44
75.31
65.78
64.58
56.53
56.05
54.58
50.99
40.14
38.38
37.61
31.94
31.13
29.47
29.19
28.60
28.37
23.15
22.81
f1 (ppm)

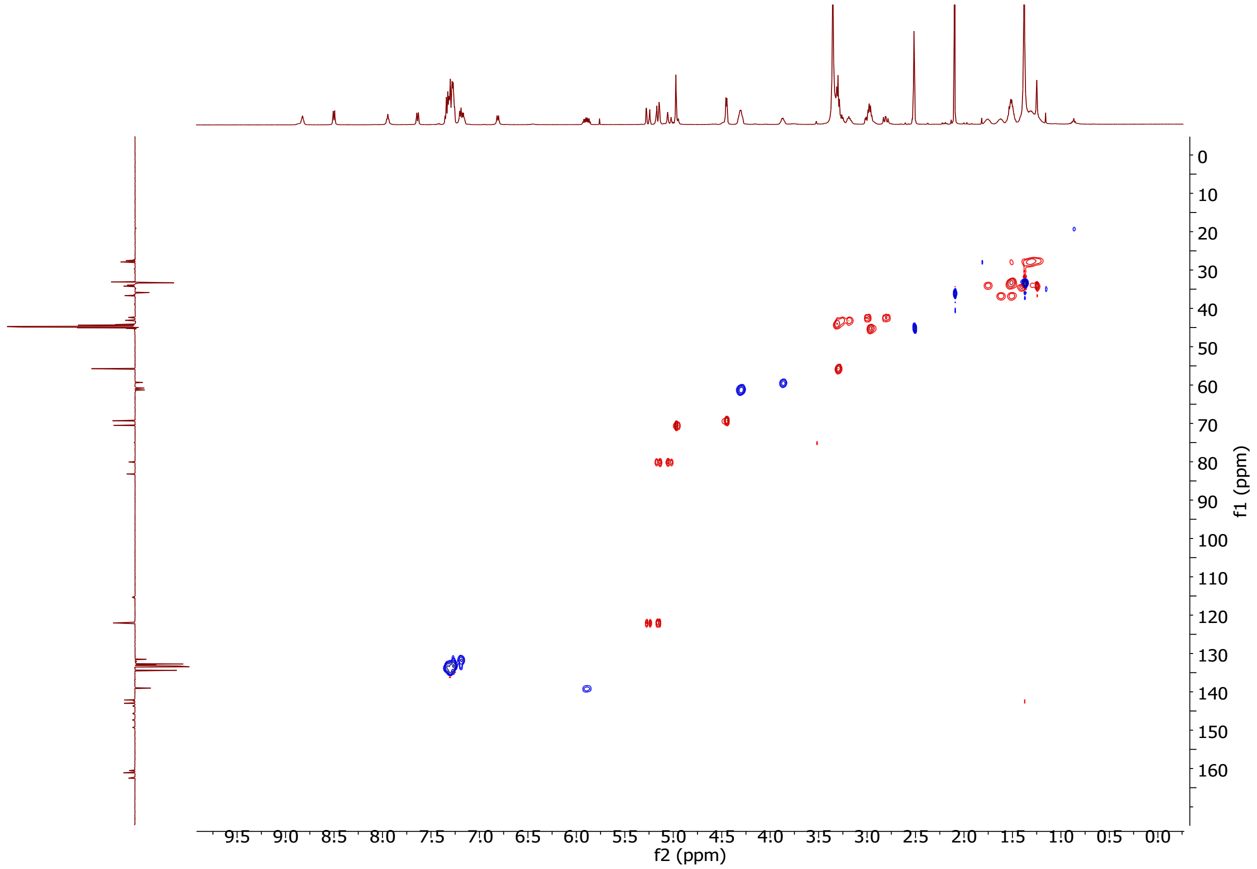

0
10
20
30
40
50
60
70
80
90
100
110
120
130
140
150
160
f1 (ppm)
9.5 9.0 8.5 8.0 7.5 7.0 6.5 6.0 5.5 5.0 4.5 4.0 3.5 3.0 2.5 2.0 1.5 1.0 0.5 0.0
f2 (ppm)

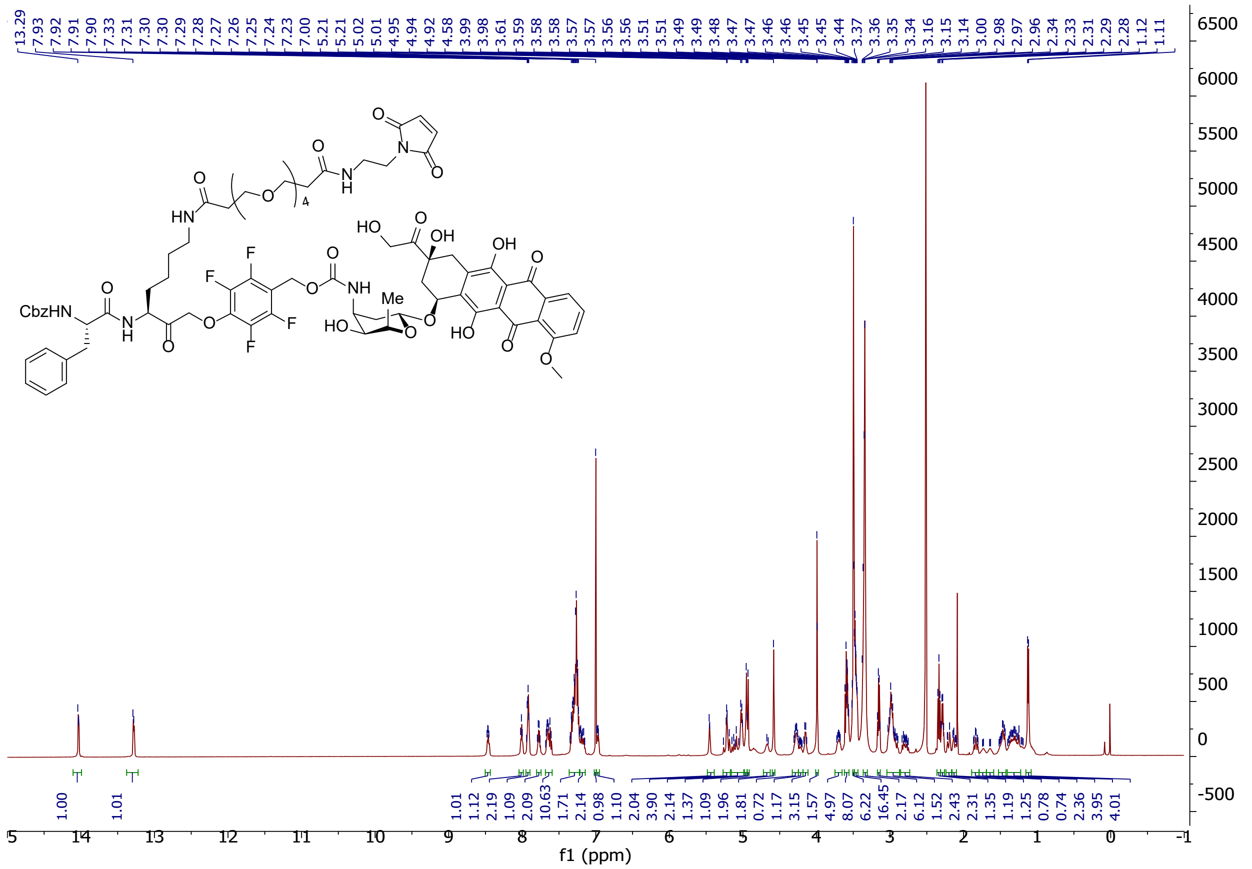

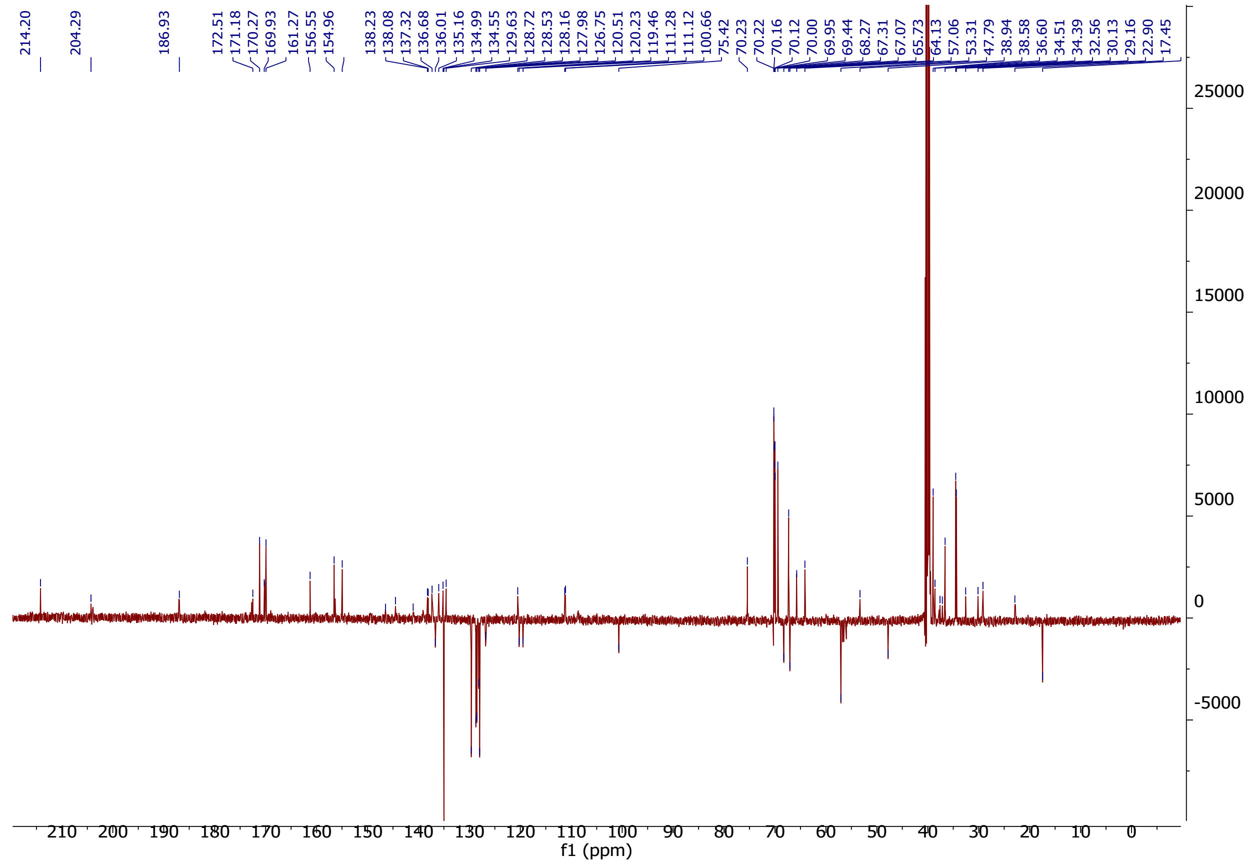

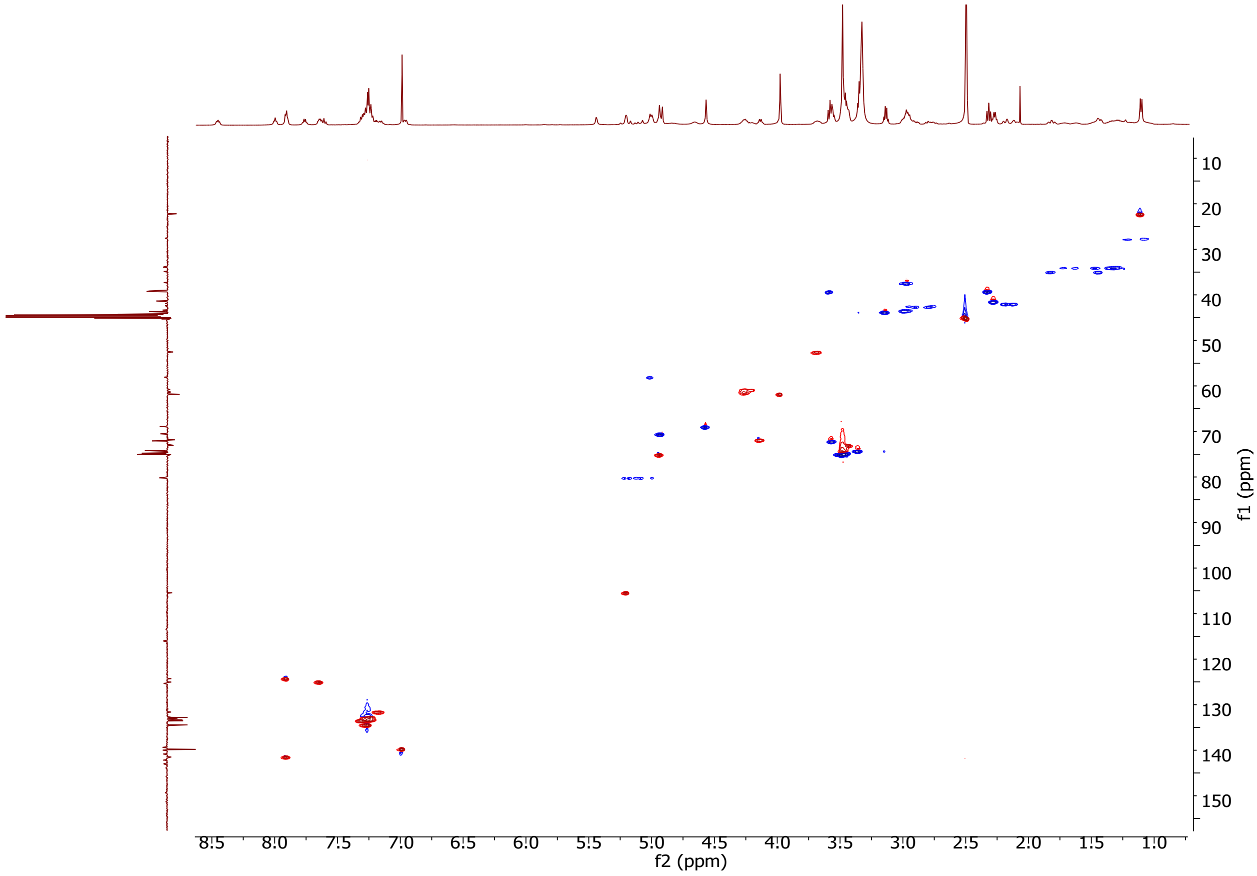

f2 (ppm)
f1 (ppm)

Supplementary References

[1] A. Emadi, R. J. Jones, R. A. Brodsky, *Nat Rev Clin Oncol* **2009**, *6*, 638-647.

[2] N. Zhang, Y. Yin, S. J. Xu, W. S. Chen, *Molecules* **2008**, *13*, 1551-1569.

[3] S. Ghosh, *Bioorg Chem* **2019,** *88*, 102925.

[4] K. J. Pienta, *Seminars Oncol* **2001**, *28*, 3-7.

[5] N. Khalesi, S. Korani, M. Korani, T. P. Johnston, A. Sahebkar, *Inflammopharmacology* **2021**, *29*, 1291-1306.

[6] E. Mini, S. Nobili, B. Caciagli, I. Landini, T. Mazzei, *Ann Oncol* **2006**, *17*, 7-12.

[7] P. A. Speth, Q. G. van Hoesel, C. Haanen, *Clin Pharmacokin* **1988**, *15*, 15-31.

[8] F. J. van Dalen, M. Verdoes, *ChemRxiv* **2022**, doi: 10.26434/chemrxiv-2022-hhb6c.

[9] T. Kitamura, B. Z. Qian, D. Soong, L. Cassetta, G. Sugano, Y. Kato, J. Li, J. W. Pollard, *J Exp Med* **2015**, *212*, 1043-1059.
